# Sustainable valorization of mango peel waste by extracting bioactive compounds for functional applications

**DOI:** 10.1038/s41598-025-28141-z

**Published:** 2025-12-07

**Authors:** Ibrahim Amin Ibrahim, Hamdy Mostafa Mohamed Ebeid, Yehia Abd El-Razik Heikal, Hesham Mohsen Elhariry

**Affiliations:** Department of Food Science, Faculty of Agriculture, Ain Shams University, Cairo, Egypt

**Keywords:** Mango peel waste, Ethanolic extraction, Supercritical CO₂ extraction, Nanoencapsulation, Multidrug-resistant (MDR) bacteria, Antioxidant, Antibacterials, Antibiofilm, Biochemistry, Biotechnology, Chemistry, Microbiology, Nanoscience and technology, Plant sciences

## Abstract

Mango peel waste is a rich source of bioactive compounds with potential for sustainable valorization. In this study, coarse and fine mango peel powders were subjected to ethanolic and supercritical CO₂ extraction. The resulting extracts were evaluated for phenolic and flavonoid content, antioxidant activity (DPPH and FRAP), as well as antibacterial, antiadhesion, and antibiofilm properties against six multidrug-resistant foodborne pathogens. The ethanolic extract from fine mango peel powder (EMPF) exhibited the highest phenolic (2488 µg GA eq/mL) and flavonoid (1282 µg rutin eq/mL) content, with quercetin (63.75 mg/L) and p-hydroxybenzoic acid (28.68 mg/L) as major compounds, and showed strong antioxidant activity (RSA = 40%, FRAP = 0.804). To enhance stability and bioactivity, EMPF was nanoencapsulated in calcium alginate (N-EMPF), yielding a favorable particle size (~ 233 nm), zeta potential (+ 23.6 mV), and encapsulation efficiency (56%). Nanoencapsulation improved antioxidant activity (RSA = 49%, TFC = 1566 µg rutin eq/mL) and significantly enhanced antimicrobial, antiadhesion, and antibiofilm effects, while maintaining low cytotoxicity in human oral epithelial cells (IC₅₀ > 100 µg/mL; viability > 70%). These findings demonstrate that nanoencapsulated ethanolic mango peel extracts represent safe, multifunctional, and sustainable sources of antioxidant, antibacterial, and antibiofilm agents with promising potential for biotechnological applications.

**Supplementary Information:**

The online version contains supplementary material available at 10.1038/s41598-025-28141-z.

## Introduction

The widespread issue of food loss and waste has become a central concern due to its profound impacts on food availability, human health, and environmental sustainability. According to the Food and Agriculture Organization (FAO), nearly 50% of fruits and vegetables are lost annually, primarily due to postharvest losses during storage, transportation, and marketing, contributing significantly to overall food loss and waste^1^. This waste not only results in the loss of edible produce but also leads to the unnecessary consumption of critical resources such as soil, groundwater, irrigation water, fertilizers, agrochemicals, energy, labor, time, and financial investments^2^. The economic impact is substantial, with annual losses from fruit and vegetable waste estimated at around US$750 billion^1^. Approximately 1.3 billion metric tons of food are wasted globally each year, with fruits and vegetables accounting for the highest proportion of this waste^3^. The fruit and vegetable processing industry is a significant contributor to this issue, generating substantial volumes of by-products such as peels, seeds, pomace, rinds, and shells, which are often discarded without further utilization (Oyom and Tahergorabi,. Exploring environmentally friendly strategies to reduce waste in the postharvest supply chain is therefore essential for promoting sustainable development and ensuring global food security.

Egypt is one of the major mango-producing countries, with annual production reaching approximately one million tons. However, a significant portion of this yield is wasted during industrial processing. Specifically, 40–60% of mangoes include inedible kernels, seeds, and peels, which are often discarded without proper treatment, contributing to both environmental degradation and economic inefficiency^4^. The seasonal nature of mango production leads to overproduction during peak harvest periods and scarcity during off-seasons, further intensifying the waste problem^5^. For instance, in 2016, Egypt produced over 600,000 tons of mangoes, yet a large portion of this output was not effectively utilized, resulting in considerable waste^4^.

Despite being classified as waste, mango by-products are rich in bioactive compounds with significant health-promoting properties. These include antioxidants, vitamins, and minerals such as carotenoids, phenolic compounds, and vitamins A, C, and E^6^. Mango seeds, especially the kernel, are a rich source of bioactive compounds, including polyphenols, flavonoids (such as mangiferin and quercetin), phenolic acids, and phytosterols. These compounds offer antioxidant, antimicrobial, antidiabetic, and anticancer properties. In addition to its therapeutic potential, the seed kernel contains essential nutrients like carbohydrates, proteins, fats, and dietary fiber, making it suitable for use in functional foods and nutraceuticals. Its health-promoting properties support its application in preventing chronic diseases and enhancing overall wellness^7^.

Mango peel constitutes 7%–24% of the total fruit weight and is a valuable source of therapeutic and functional bioactive compounds. In particular, it contains key components such as polyphenols, carotenoids, mangiferin, flavonoids, catechin, phenolic acids, and gallic acid derivatives^8^. These compounds exhibit antioxidant, anti-inflammatory, and anticancer activities^6,7^. Mango peel is exceptionally rich in distinctive bioactive compounds such as mangiferin and specific gallotannins (e.g., penta-O-galloyl-glucose), which are rarely present in other fruit peels^8^. The nutraceutical and pharmaceutical significance of mangiferin, a xanthone polyphenol, has been widely documented for its antioxidant, anti-inflammatory, antidiabetic, and anticancer effects, as well as its cardio-, hepato-, and gastroprotective properties^9^. Comparative studies on tropical fruit peels further revealed that mango peel exhibits the highest total phenolic content and strongest antioxidant activity, attributed to its abundance of gallic acid, quercetin, chlorogenic acid, and mangiferin^10^. The synergistic action of these phytochemicals supports the use of mango peel and its by-products as valuable functional food ingredients or nutraceuticals with broad cytoprotective and antimicrobial benefits. Pomegranate peels for natural colorant production are well established, while citrus peels are primarily utilized for pectin and essential oil extraction^11^. Overall, mango peel represents the most promising and sustainable candidate for industrial valorization, aligning with circular economy principles by transforming agro-industrial waste into health-promoting functional resources.

Multidrug resistance (MDR) in bacteria refers to the ability of bacterial strains to withstand the effects of multiple antibiotics, often from three or more different classes, making infections increasingly difficult to treat and control^12^. MDR can arise from the accumulation of resistance genes on plasmids, transposons, or chromosomes. Plasmid-mediated resistance has become more common since the introduction of antibiotics^13^.

Widespread antibiotic application in food chain production can lead to the development of resistant microorganisms^14^. These resistant strains may spread to humans through direct contact, environmental pathways, or consumption of animal-derived products, posing serious public health concerns. As a response to this issue, incorporating natural alternatives to control microbial growth is emerging as a promising strategy to combat antibiotic resistance. Additionally, mango peel extracts have demonstrated antibacterial properties against both Gram-positive and Gram-negative bacteria, making them suitable for antimicrobial applications^15^. To harness the potential of mango waste, advanced extraction techniques are employed to improve the bioaccessibility and yield of bioactive compounds. These include solvent extraction, supercritical fluid extraction (SCFE), ultrasound-assisted extraction, and microwave-assisted extraction. SCFE offers several advantages, such as high selectivity, environmental friendliness, and efficiency. The use of co-solvents like ethanol enhances the extraction of specific compounds such as carotenoids and phenolics^16^. In thermodynamic optimization studies, the supercritical CO₂ extraction parameters (300 bar and 50 °C) had demonstrated maximum extraction yield for polyphenols. This is due to a balance between increased CO₂ density (which enhances solvating power and extraction efficiency) and decreased diffusivity (which can reduce interaction with the solute). At higher pressures (more than 300 bar), there is no further increase in yield and may even reduce it due to lower diffusivity^17^. Moreover, Gallic acid was extracted at its maximum level when the parameters were 300 bar and 50 °C. Ethanol is considered a green solvent, safe for use in pharmaceutical and food industries, and is preferred over other solvents due to its safety profile^18^. Using ethanol at concentrations up to 50% significantly improves the yield of flavonoids from mango peels^19^. Ethanolic extraction is efficient, safe, and environmentally friendly, making it ideal for recovering phenolics and flavonoids from mango peel^20^.

Nanotechnology is increasingly applied in food science to enhance the stability, bioavailability, and controlled release of bioactive compounds. Nanoencapsulation protects sensitive compounds from environmental factors such as temperature, light, pH, and oxygen^21^. This technology improves the functionality of bioactive compounds and facilitates their incorporation into food systems. Nanoencapsulation is used to deliver vitamins, antioxidants, enzymes, and antimicrobials in functional foods, nutraceuticals, and dietary supplements, enhancing their therapeutic efficacy^22^. This approach aligns with circular economic principles by transforming agricultural waste into high-value functional ingredients. Therefore, this study aims to valorize mango processing waste, particularly mango peel, by extracting and nanoencapsulating bioactive compounds using environmentally friendly and advanced technologies. Antioxidants, antibacterial, antiadhesion, and antibiofilm activities targeting multidrug-resistant (MDR) foodborne pathogens were also considered. This goal contributes to establishing sustainable approaches for transforming mango waste into functional ingredients applicable in the food, pharmaceutical, and nutraceutical industries.

## Materials and methods

Mango peels from the *Zebda* variety were collected after mango pulp processing, from one batch in season 2022, at Orion Food Industries Company, located in 6th of October City, Egypt.

### Chemicals

The following chemicals were used in the present study: Folin–Ciocalteu reagent (Oxford laboratory, India), ferric chloride (FeCL_3_) and sodium alginate (Alpha Chemika, India), anhydrous aluminum chloride (SD Fine-Chem Limited), sodium nitrate (Advent Chembio, India), gallic acid (Fisher Scientific, UK), and rutin, (Loba Chemie Pvt. Ltd., India), 2,2-diphenyl-1-picrylhydrazyl radical (DPPH^•^) and 2,4,6-tris (2-pyridyl)-s-triazine (TPTZ) (Sigma Chemicals, USA), crystal violet and safranin (Merck, Germany) and the other chemicals (El-Gomhouria Company for Trading Pharmaceuticals, Chemicals and Medical Supplies, Cairo, Egypt). All used chemicals and reagents were of analytical grade.

### Bacterial strains

Three Gram-positive pathogenic bacterial strains, including *Bacillus cereus* ATCC 1076, *Listeria monocytogenes* ATCC 7644, and *Staphylococcus aureus* ATCC 653P, and three Gram-negative pathogenic bacterial strains, including *Escherichia coli* ATCC 10,536, *Salmonella* Typhimurium ATCC 14,028, and *Campylobacter jejuni* subsp. *jejuni* CCM 6214, were obtained from the Egyptian Microbial Culture Collection (EMCC) at Cairo Microbial Resources Center (MIRCEN), Faculty of Agriculture, Ain Shams University, Egypt.

### Culture media and antibiotics

Brain Heart Infusion (BHI) broth (Merck, Germany), Nutrient agar, Mueller-Hinton agar (MHA), and Tryptic Soy Broth (TSB) were used for microbiological experiments. Novobiocin (NV, 5 µg), Chloramphenicol (C, 30 µg), Oxacillin (OX, 5 µg), Streptomycin (S, 10 µg), and Penicillin-G (P, 10 µg) were purchased from HiMedia Laboratories, India. Norfloxacin (NOR, 10 µg), Cefadroxil (CFR, 30 µg), Pefloxacin (PEF, 5 µg), Piperacillin (PRL, 100 µg), and Flucloxacillin (FL, 5 µg) were obtained from Bioanalyses Tıbbi, Türkiye.

### Preparation of mango peel powder

Mango peels were washed with tap water, drained, and dried at 50 °C for 18 h using a hot air oven (DHG-9140 A, China) as described by^23^. The dried peels were ground using a hammer mill, followed by fine grinding using a Mienta Grinder Presto (Model CG44126B, Mienta, China). The resulting powder was sieved through a 0.5 mm sieve at a speed of 8 for 10 min (RETSCH sieve shaker-GDR) to separate it into two particle sizes: coarse (≥ 0.5 mm) and fine (≤ 0.5 mm). The powders were then stored at freezing temperature.

### Proximate analysis

Mango peel powder coarse (MPC) or mango peel powder fine (MPF) samples were ground to pass through a 0.25 mm screen before analysis. Laboratory measurements included major constituents, and additionally, calcium and phosphorus. Analyses were in accordance with the methods issued by VDLUFA (dry matter: VDLUFA III 3.1; crude protein: VDLUFA III 4.1.1 modified according to macro-N determination (Vario Max CN); crude fiber: VDLUFA IΠΙ 6.1.4; crude ash: VDLUFA III 8.1; crude fat: VDLUFA III 5.1.1; calcium and phosphorus: VDLUFA VII 2.2.2.6)^24^.

### Fourier transform infrared spectra analysis

Fourier transform infrared (FTIR) spectroscopy was used to obtain specific information about the chemical bonds and molecular structure of samples. The analysis was carried out according to the method of^25^. Spectra were recorded using a spectrometer (TENSOR-27 FTIR, Bruker Optik GmbH, Germany). Infrared spectrum was used within the range of (400–4000) cm^− 1^. FTIR PerkinElmer Spectrum IR software version 10.7.2 (Waltham, Massachusetts, United States) was applied to analyze the FTIR data.

### Ethanolic extraction of mango peel powder

Mango peels powder (MPC, coarse, and MPF, fine) was mixed with ethanol solution (50%) at a ratio of 1:50 (w/v)^19^. The mixtures were gently shaken in a water bath (Abbota,110X, USA) at 50 °C for 60 min. They were then centrifuged at 5000 rpm for 20 min using a centrifuge (Remi-r32c, India), following the method described by Dorta, et al.^26^. The extracts produced by this procedure are named EMPC and EMPF for the Ethanolic Mango Peel powder Coarse and Fine, respectively. The extracted yield was expressed from the following equation:

### Supercritical fluid extraction of mango peel powder

Supercritical fluid extraction was performed using a supercritical CO_2_ extractor (Sigmar Mothes Hochdrucktechnik GmbH, Berlin, Germany) at 300 bar and 50 °C^17,27^. A back-pressure regulator was used to control the flow and pressure in the extraction unit. The sample was placed in the extraction vessel. Ethanol, as a cosolvent, 2.1 mL/min, was supplied by a high-pressure fluid pump and mixed with the CO_2_ flow before entering the extraction cell. The supercritical fluid extraction conditions applied in the present study are presented in Table 1. The flow rate was 5.295 kg/h for MPF and 4.74 kg/h for MPC. The extract was collected in a pre-weighed collection bottle, which was wrapped with aluminum foil during and after extraction to avoid oxidation of the extract components. The extracts produced by this procedure are named SMPC and SMPF for the supercritical extracts of mango peel powder, coarse and fine, respectively. The extracted yield was expressed from the following equation and presented in Table 1:

**Table 1:** Supercritical fluid extraction conditions of mango peel powder coarse (MPC) and mango peel powder fine (MPF).

Input sample	Pressure (bar)	Temperature (°C)	Extraction time (h)	CO₂ flow rate (kg/h)	CO₂ amount passed through (kg)	Initial weight of extraction material (g)	Final weight of extraction material (raffinate)(g)	Difference between initial and final weight (extract) (g)	Collected extract in separator 1(g)
MPF	300	50	5	1.06	5.295	179.00	170.20	8.80	270.75
MPC				0.95	4.74	165.00	154.90	10.10	291.24

$$\:\text{Y}\text{i}\text{e}\text{l}\text{d}\left(\text{\%}\right)=\:\frac{\text{D}\text{i}\text{f}\text{f}\text{e}\text{r}\text{e}\text{n}\text{c}\text{e}\:\text{b}\text{e}\text{t}\text{w}\text{e}\text{e}\text{n}\:\text{i}\text{n}\text{i}\text{t}\text{i}\text{a}\text{l}\:\text{a}\text{n}\text{d}\:\text{f}\text{i}\text{n}\text{a}\text{l}\:\text{w}\text{i}\text{e}\text{g}\text{h}\text{t}\:\text{o}\text{f}\:\text{e}\text{x}\text{t}\text{r}\text{a}\text{c}\text{t}\text{i}\text{o}\text{n}\:\text{m}\text{a}\text{t}\text{e}\text{r}\text{i}\text{a}\text{l}\:}{\text{i}\text{n}\text{i}\text{t}\text{i}\text{a}\text{l}\:\text{w}\text{i}\text{e}\text{g}\text{h}\text{t}\:\text{o}\text{f}\:\text{e}\text{x}\text{t}\text{r}\text{a}\text{c}\text{t}\text{i}\text{o}\text{n}\:\text{m}\text{a}\text{t}\text{e}\text{r}\text{i}\text{a}\text{l}}\times\:100$$

### Pesticide determination using LC-MS/MS

Pesticide residues in mango peel extracts (EMPC, EMPF, SMPC, and SMPF) were determined according to the method described by Attallah, et al.^28^. High-performance liquid chromatography (HPLC) (Agilent 1200 Series) was coupled to an API 4000 Qtrap MS/MS from AB Sciex, with an electrospray ionization (ESI) interface in the positive mode, source temperature was 400 °C, and ion spray potential was 5500 V. Separation was achieved on an Agilent C18 column ZORBAX Eclipse XDB 4.6 × 150 mm with 5.0 μm particle size. The ESI source, N2 nebulizer, curtain gas, declustering potential (DP), collision energy (CE), the MRM transitions, and other parameters were optimized according to the manufacturer’s recommendations using a Harvard apparatus syringe pump to introduce individual pesticide solutions into the MS instrument. A gradient elution program was used at 500 µL/min, in which one reservoir contained 10 mM ammonium formate solution in methanol: water (1:9, v/v) and the other contained LC-MS grade methanol. The total run time was 16 min, and the injection volume was 5.0 µL. Data asset and instrument control were performed using Analyst software version 1.6.

### Production of encapsulated EMPF

The preparation of encapsulated nanosphere from mango peel extract included two steps as follows:

#### Preparation of calcium chloride nanoparticles

Calcium chloride solution (0.1 M) was dissolved in pure ethanol, then added to palm kernel oil at a concentration of 6% (150 µL) as an oil phase, with 1.5 µL of Span 80 as surfactant. The mixture was sonicated using a probe sonicator (Qsonica, LIC, U.S.A) at a constant frequency of 20 kHz for 5 min. The alcohol was evaporated under vacuum at 40 ᵒC overnight^29^.

#### Preparation of EMPF-loaded alginate nanosphere

EMPF-loaded alginate nanosphere was prepared by the methods of Paques, et al.^29^, which was modified by Rahnemoon, et al.^30^. Briefly, sodium alginate was added to the MPF extract to a final ratio of 4:1. This mixture was gradually added to an oil phase (50 mL of palm kernel oil, 35 mL of coconut oil, and 150 µL of Span as surfactant). To form a water-in-oil nanoemulsion, ultra-homogenization was applied (11500 rpm for 15 min). The alginate gelation was completed by adding calcium chloride nanoparticles dispersed in oil to the system, followed by ultra-homogenization at 11,500 rpm for 5 min. The ratio of alginate to calcium chloride nanoparticles was 1:9. The nanoemulsion was washed three times with acetone to remove the oil phase and unreacted particles by centrifugation at 10,000 rpm/4ᵒC for 5 min. The pellet of encapsulated EMPF nanoparticles (N-EMPF) was resuspended in 100 mL of deionized water and stored at 4ᵒC until use.

### Characterization of N-EMPF

Characterization of N-EMPF was carried out using the Microtrac Nanotrac Wave II analyzer, which applies Dynamic Light Scattering (DLS) enhanced with Reference Beating Technology for increased sensitivity. Measurements included particle size, Zeta potential, and polydispersity index (PDI). Zeta potential was recorded. All measurements were conducted at ~ 22 °C using the FLEX software, with input parameters including dispersant viscosity and dielectric constant. Analyses were performed in triplicate, and all sample cells were thoroughly cleaned before and after each run. The PDI was calculated by using the following equation: PDI = σ/µ, where σ is the standard deviation (width of the size distribution) and µ is the mean diameter of the nanoparticles^31^.

### Determination of encapsulation efficiency (EE)

The encapsulation efficiency (EE) value was determined based on the method described by^32^. Here, EE was calculated based on the changes in total bioactive components after encapsulation. The EE was determined by the following equation:

### Cytotoxicity

Cytotoxicity of EMPF and N-EMPF was studied at Global Research Labs, 3 (Medical Center 2, Nasr City, Cairo, Egypt). EMPF and N-EMPF were provided in powder form. The method applied was described in detail by^33^. Briefly, a stock solution of 100 µg/mL was prepared by reconstitution of 0.1 g in the appropriate volume of 1 mL of dH_2_O, followed by sonication for 5 s, aliquoted, and stored at − 20 °C until use. For all experiments, the final concentrations of the test compound were prepared by diluting the stock with the medium. The carrier solvent (0.1% DMSO) was added to the control cells. The half-maximal cytotoxic effect (IC_50_) of different EMPF formulas on normal Human Oral Epithelial cells (h-OECs, ACCEGEN, USA) was determined. The h-OECs were prepared for experiments using the conventional trypsinization procedure with trypsin/EDTA. Cells were seeded on a 96-well culture plate. 8 × 10^3^ cells per well were seeded in 200 µL of Dulbecco’s Modified Eagle Medium (DMEM), supplemented with 10% Fetal Bovine serum (FBS), and 1% of penicillin G sodium (10.000 UI), streptomycin (10 mg), and amphotericin B (25 µg) (Gibco, ThermoScientific, Germany). Culture plates were incubated at 37 °C in an atmosphere of 5% CO_2_ for 24 h to attach cells. On the next day, a constant concentration of serial concentrations of each formula, 0.01, 0.1, 1.0, 10, 100 µg/mL, was prepared. In addition, the carrier solvent (0.1% DMSO) was used for control cells. Cells were maintained at 37 °C in an atmosphere of 5% CO_2_ for 48 h. At the end of incubation, the cell cytotoxicity assay was performed using the Vybrant^®^ MTT Cell Proliferation Assay Kit, cat no: M6494 (Thermo Fisher, Germany). A 100 µL of media was removed and replaced with new media. Twenty µL of 4,5-dimethylthiazol-2-yl)−2,5-diphenyltetrazolium bromide (MTT) solution (1 mg/mL) (Invitrogen, ThermoScientific, Germany) was added to each well. The plates were incubated at 37 °C and 5% CO2 for four hours. Finally, the MTT solution was removed, and 100 µL of sodium dodecyl sulfate with hydrochloric acid (SDS-HCL) was added to the wells. Cell viability was determined by measuring the optical density at 570 nm using a spectrophotometer (ELx 800; Bio-Tek Instruments Inc., Winooski, VT, USA).

### Total phenolic content

The total phenolic content (TPC) of ethanolic extracts, supercritical fluid extracts, and MPF-loaded nanoparticles was determined spectrophotometrically using the modified Folin–Ciocalteau colorimetric method^34^. A portion of the tested extract (0.5 mL) was mixed with 2.5 mL of Folin-Ciocalteau reagent 10% in a test tube and allowed to stand for 6 min at 37 °C, then 2.5 mL of 7.5% Na2CO3 solution was added and incubated at 37 °C for 30 min. The absorbance was measured at 765 nm using a spectrophotometer (Jenway 6105, UK). The TPC was expressed as µg gallic acid equivalent (GAeq)/mL sample using the following equation based on the calibration curve: Y = 0.0024x – 0.0563; R^2^ = 0.999.

### Total flavonoid content

Total flavonoid content (TFC) of extracts and nanoparticles was determined spectrophotometrically using the modified method previously described by Chang, et al.^35^. The standard solutions of quercetin (0.5, 1, 10, and 25 mg/100 mL) were prepared in 95% methanol. One mL of the extracts or quercetin standard solution was mixed with 4 mL of distilled water and 0.3 mL of 5% sodium nitrate (NaNO₂) solution. After 5 min of holding, 0.3 mL of 10% aluminum chloride (AlCl₃) solution was added, and the mixture was gently mixed on a Vortex mixer (VELP Scientifica 0.0171, Italy) for 6 min. Two mL of 1 M sodium hydroxide (NaOH) was added, and the final volume was adjusted to 10 mL with distilled water. The absorbance was measured at 510 nm against a prepared blank using a spectrophotometer (Jenway 6105, UK). The TFC was expressed as [µg Rutin eq/mL sample], based on the standard calibration curve (Y = 0.0007x + 0.0037; R² = 0.999). Data are represented as means ± SD.

### DPPH^•^ radical scavenging activity

Radical scavenging activities (RSA) of extracts and nanoparticles were tested by the spectrophotometric method of^36^. using the DPPH˙. A 20 µL of different extracts was added to 2 mL of freshly prepared methanolic DPPH^•^ solution (0.00374 g/L methanol). The reaction was monitored by reading absorbance at 517 nm after 30 min holding using a spectrophotometer (Jenway 6105, UK). The scavenged percent of DPPH^•^ in the reaction was calculated according to the following equation: $Inhibition~\left( \% \right)~ = ~A_{ \circ } - ~A~/~A_{ \circ } ~ \times ~100$

Where $A_{ \circ }$ was the absorbance of the control, and *A* was the absorbance of the sample.

### Ferric ion reducing antioxidant power (FRAP) assay

The FRAP methodology was described by^37^. The following stock solutions were prepared: A 0.03 M acetate buffer solution, a 10 mM TPTZ solution, and a 20 mM ferric chloride solution. The fresh working solution was prepared by mixing the acetate buffer solution, TPTZ solution, and ferric chloride solution in a 10:1:1 ratio, respectively, under darkness. For each analysis, 25 µL of the blank, standard, or sample extracts and nanoparticles were mixed with 2 mL working solution and mixed gently. The mixture was incubated at 37 °C for 30 min. The absorbance was measured at a wavelength of 593 nm using a spectrophotometer (Jenway 6105, UK). The FRAP is expressed as optical density.

### Antibacterial activity

Antibacterial activities of extracts and nanoparticles were evaluated using a well diffusion method. MHA plates were inoculated with 0.1 mL (10^6^ cfu/mL) of the bacterial strains. Wells of 5 mm diameter were cut into MHA plates using a sterilized corkborer (5 mm). Subsequently, 80 µL of each extract was added to each well. The plates were incubated at 37 °C for 24 h^38^. For comparison, the susceptibility of bacterial strains to ten different antibiotics was tested using the Kirby-Bauer disk diffusion method, following the procedure described by Bauer et al.^39^ The diameters of the zones of inhibition were measured in millimeters.

### Antiadhesion and antibiofilm activity

To study the anti-biofilm effect of the extracts, the standard method described by^40^ and modified by^41^ was used, with the modification of adding the studied extracts to the wells in microtiter plates. Briefly, sterile 96-well polystyrene microtiter plates were filled with 230 µL of tryptone soy broth (TSB), followed by the addition of 50 µL of extracts or nanoparticles and 20 µL of each bacterial culture to the designated wells. Negative control wells contain TSB only. The plates were incubated aerobically at 37 °C for 6 h (for determining antiadhesion) and 24 h (For determining antibiofilm) effects. The contents of the microtiter plates were poured off, and the wells were washed three times with 300 µL of P-buffer. The remaining attached bacteria were fixed with 250 µL of methanol per well. After 15 min microtiter plates were emptied and air-dried. The microtiter plates were stained with 250 µL per well of 1% crystal violet used for Gram staining for 5 min. The excess stain was removed by gently rinsing the microtiter plates under running tap water. Following air drying, the dye retained by the adherent bacterial cells was extracted by adding 250 µL of a solution composed of 80% ethanol and 20% acetone to each well. The absorbance of each well was measured at 630 nm^42^ using a microplate ELISA reader (DIReader ELx800, DIALAB GmbH, Austria), the cut-off absorbance (*Ac*) was the mean absorbance of the negative control. Strains were classified as follows: *A* = *Ac* = no biofilm producer (0); *Ac* < *A* = (2 x *Ac*) = weak biofilm producer (+); (2 x *Ac*) < *A* = (4 x *Ac*) = moderate biofilm producer (++); (4 x *Ac*) < *A* = strong biofilm producer (+++). All tests were carried out in triplicate, and the results were averaged.

### Statistical analysis

Statistical analysis was performed using Minitab software (version 21.2). A one-way analysis of variance (ANOVA) was applied to evaluate significant differences among treatments at a probability level of *p* ≤ 0.05. When the ANOVA indicated significance, Tukey’s Honestly Significant Difference (HSD) post-hoc test was conducted to determine pairwise differences between multiple treatments.

## Results and discussion

### Proximate analysis

Mango peels were characterized by their high content of crude fiber and non-fibrous carbohydrates, where the obtained percentages on a dry matter basis were 15.25 and 72.31%, respectively (Table 2). This result aligns with the findings of^43^, where the protein content measured was 6.44%, which is notably higher than the values reported in earlier studies. They stated that the range of protein content in mango peel was 1.5 to 6.6%. Fat and ash contents in the studied mango peel were 2.4 and 3.6%, respectively. These values were in the previously mentioned range in mango peel for fat (1.6% to 3.7%), and ash content (1.2 to 4.2%)^44^.

**Table 2:** Proximate analysis of mango peels.

Components (%)	Mango peels
Moisture	10.13
Dry matter	89.87
Major components (%) from dry matter	
Protein	6.44
Fat	2.40
Ash	3.60
Crude fibers	15.25
Non-fibrous carbohydrates^*^	72.31
Phosphorus	0.12
Calcium	0.27

^*^ was calculated from this equation [100 – (moisture + protein + fat + ash + crude fiber)].

### FTIR spectroscopy of mango peel powder

**Fig. 1:**
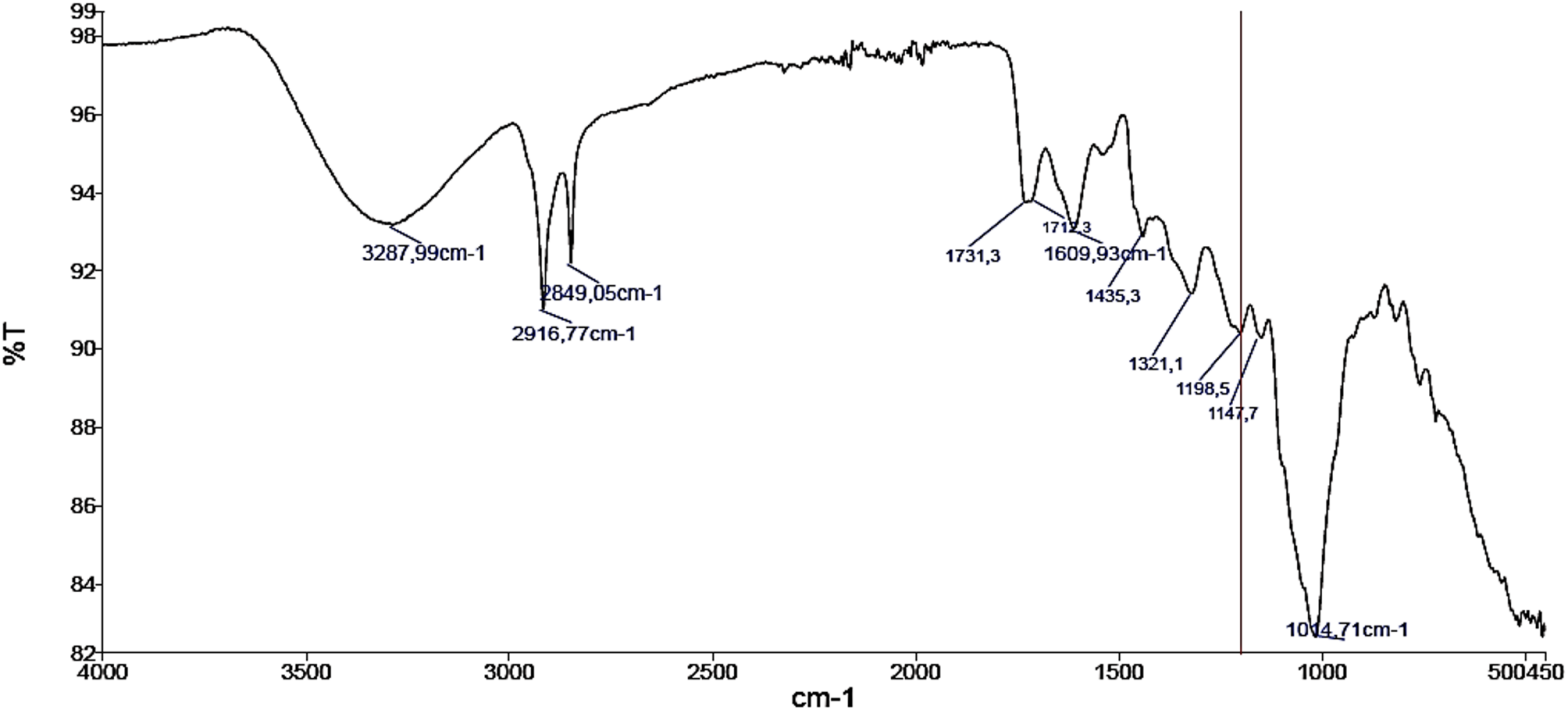
FTIR spectra of mango peel powder.

The FTIR spectrum of mango peel shows absorption peaks from 4000 to 450 cm⁻¹, which correspond to various functional groups commonly associated with bioactive compounds present in the mango peel matrix (Fig. 1). The broad peak at 3287 cm⁻¹ is primarily associated to the O-H stretching vibrations of water, cellulose, and carbohydrates, as well as potential contributions from N-H stretching of proteins^45^. The peaks at 2849 cm⁻¹ and 2916 cm⁻¹ are associated with the C-H stretching vibrations of methyl and methylene groups, indicating the presence of various organic compounds, including cellulose, hemicellulose, lignin, carbohydrates, and fatty acids. These findings are supported by the observed FTIR spectra and the known chemical composition of plant materials^45^. The vibration peaks at 1731 cm⁻¹ and 1712 cm⁻¹ are primarily associated with carbonyl (C = O) groups, which are found in various organic compounds, such as gallic acid and mangiferin^46^. Additionally, the presence of organic acids and sugars in mango peel also contributes to these absorption peaks. The peak at 1609 cm⁻¹ was likely associated with aromatic C = C bonds, which are common in polyphenols and flavonoids found in mango peel^20^. These compounds include mangiferin, quercetin, and gallic acid. Moreover, peaks in the range of 1435.3–1321.1 cm⁻¹ are associated with C–H bending vibrations (–CH₂, –CH₃), symmetric COO^−^ stretching, and C–O stretching, indicating the presence of organic acids, sugars, and phenolic compounds^20^. Peaks between 1198.48 and 1014.71 cm⁻¹ correspond to C–O–C and C–O stretching vibrations typical of glycosides, alcohols, flavonoids, and polysaccharides^20^. In general, mango peel could be considered as an important source of bioactive components due to its rich content of polyphenols, flavonoids, organic acids, sugars, and glycosides, as revealed by FTIR spectral analysis, showing diverse functional groups such as O–H, C–H, C = O, C = C, and C–O–C, which are characteristic of health-promoting phytochemicals.

### Mango peel extracts

**Fig. 2:**
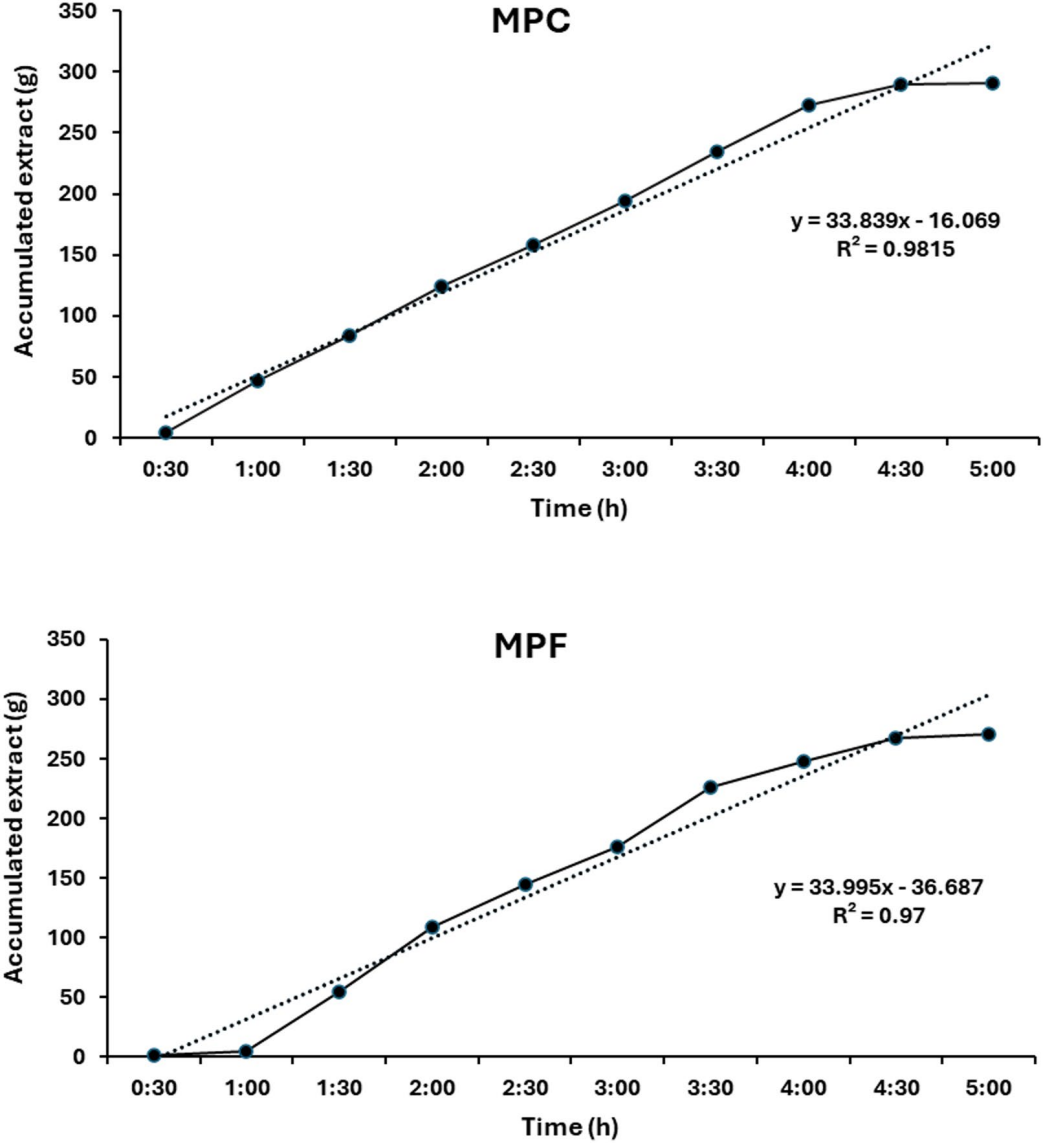
Supercritical fluid extraction kinetics of coarse (MPC) and fine (MPF) mango peel powder.

Bioactive compounds were extracted by two methods: ethanol extraction and supercritical fluid extraction. Figure 2 illustrates the extraction kinetics of bioactive compounds from fine and coarse mango peel powder, a fruit processing waste, using supercritical CO₂ extraction. The results show that fine powder yields a slightly higher extraction efficiency over time, with an extraction rate (ER) of 33.995 g/h compared to 33.839 g/h for coarse powder. This modest improvement is attributed to the increased surface area, reduced diffusion resistance, and enhanced mass transfer associated with smaller particle sizes. The nearly linear extraction profiles for both samples suggest a constant extraction rate phase, reflecting effective solute-solvent interaction under the applied supercritical CO₂ conditions. These findings align with previous studies that emphasize the role of particle size in enhancing the efficiency of supercritical CO₂ extraction by improving solvent penetration and matrix disruption^47^. Therefore, particle size is a critical parameter in optimizing supercritical CO₂ extraction for the recovery of valuable bioactive compounds from agro-industrial waste such as mango peel.

### Pesticide residuals in mango peel extracts

Before using the extracts, it was necessary to ensure they were free of pesticide residues. The results of pesticide residue analysis in mango peel extract (EMPC, EMPF, SMPC, and SMPF) showed that all samples were completely free of any detectable residues at the limit of detection of 0.01 mg/kg. The assay covered a wide range of chemical pesticide classes, exceeding 70 diverse categories (337 pesticides), including commonly used compounds such as Organophosphorus, Carbamate, Azole, Neonicotinoid, and Pyrethroid (supplemented data, S1). This finding indicates that the mango peel extract possesses a high level of purity from pesticide residues, which enhances its potential applicability in food-related fields. Despite the comprehensive coverage of chemical classes in the analysis, no pesticide residues were detected, serving as a strong indicator of the extract’s safety and quality.

### Bioactive components and antioxidant activity of mango peel extract

Among the four mango peel extracts evaluated (SMPC, SMPF, EMPC, and EMPF), the EMPF demonstrated the most potent antioxidant profile (Table 3). The extraction yield of bioactive compounds from mango peel powder varied notably depending on both the extraction technique and the particle size of the material (Table 3). The ethanolic extraction exhibited significantly higher yields compared to the supercritical CO₂ extraction. The fine ethanolic extract (EMPF) achieved the highest yield of 33.0%, followed by the coarse ethanolic extract (EMPC) with 23.2%, indicating that the smaller particle size improved solvent penetration and mass transfer efficiency^48^. In contrast, the supercritical extracts showed relatively lower yields, with SMPC (6.1%) and SMPF (4.9%), These findings are consistent with previous reports indicating that Soxhlet extraction using ethanol from dried mango peels achieved the highest yield (13–59%)^49^.

**Table 3:** Bioactive components and antioxidant activity of mango peel extracts.

Extract	Extraction yield^*^ (%)	Antioxidant activity		bioactive compounds		
		DPPH RSA%	FRAP OD_593_	TPC µg GA eq/mL	TFC µg Rutin eq/mL	Total µg/mL
SMPC	6.1	31^c^ ± 0.55	1.133^a^ ± 0.01	197^d^ ± 9	701^c^ ± 63	898
SMPF	4.9	33^c^ ± 2.68	0.796^b^ ± 0.12	324^c^ ± 23	98^d^ ± 63	422
EMPC	23.2	37^b^ ± 2.68	0.860^b^ ± 0.03	1799^b^ ± 72	137^d^ ± 7	1936
EMPF	33	40^ab^ ± 2.19	0.804^b^ ± 0.12	2488^a^ ± 47	1282^b^ ± 74	3770
N-EMPF		49^a^ ± 1.56	0.896^b^ ± 0.08	80^e^ ± 4	1566^a^ ± 1	1646
EE						56%

*DPPH* 2,2-diphenyl-1-picrylhydrazyl, *RSA* Radical scavenging activities, *FRAP* Ferric ion reducing antioxidant power, *TPC* Total phenolic content, *TFC* Total flavonoid content, *SMPC* Supercritical extracts of mango peel coarse ‎powder, *SMPF* Supercritical extracts of mango peel fine ‎powder, *EMPC* Ethanolic extract of mango peel coarse powder, *EMPF* Ethanolic extract of mango peel fine powder, *N-EMPF* Nonencapsulated EMPF, *EE* encapsulation efficiency. ^*^ Extraction yield% was calculated by subtracting the difference between the initial and final dry weight of extracted materials from the initial dry weight, as described in the method section.

whereas supercritical CO₂ extraction resulted in the lowest yield (around 6%) according to^50^. The difference in yield between the two methods can be attributed to the polarity of the extraction medium; being a polar solvent, it is more efficient in extracting phenolic and flavonoid compounds, whereas supercritical CO₂ primarily targets less polar constituents^51^.

The antioxidant activity and bioactive compound content of mango peel extracts were evaluated using DPPH radical scavenging activity (RSA), ferric reducing antioxidant power (FRAP), total phenolic content (TPC), and total flavonoid content (TFC) (Table 3). Among the tested extracts, N-EMPF exhibited the highest RSA% (49 ± 1.56%), indicating a strong free radical scavenging capacity, followed by EMPF (40 ± 2.19%) and EMPC (37 ± 2.68%). The lowest RSA was observed in SMPC and SMPF, which showed statistically similar values (31–33%). Interestingly, the highest TPC was recorded in EMPF (2488 ± 47 µg GA eq/mL), significantly surpassing all other extracts, while N-EMPF showed a notably lower TPC (80 ± 4 µg/mL). However, N-EMPF exhibited the highest TFC (1566 ± 1 µg Rutin eq/mL), suggesting that its strong antioxidant activity may be primarily attributed to its rich flavonoid content rather than phenolics alone. These results are consistent with recent findings that ethanol is an effective solvent for extracting polyphenols and flavonoids from fruit byproducts, including mango peels, due to its polarity and ability to penetrate plant cell walls^52^. Although FRAP values showed less variability between ethanolic and supercritical extracts, SMPC demonstrated the highest ferric reducing power (1.133 ± 0.01 OD_*593*_), which was significantly different from the rest. Overall, EMPF and N-EMPF presented distinct antioxidant profiles, reflecting their enrichment in various classes of phytochemicals, and highlighting the impact of fractionation and extraction methods on the functional properties of mango peel extracts.

High-performance liquid chromatography (HPLC) analysis of the EMPF revealed that quercetin was the predominant phenolic compound, present at a concentration of 63.75 mg/L, followed by p-hydroxybenzoic acid at 28.68 mg/L (supplemented data, S2). These findings are in agreement with recent studies that have identified quercetin as one of the most abundant flavonoids in mango peel. For instance, Suleria, et al.^53^ reported that mango peel contains significantly high levels of quercetin (11.9 ± 0.4 mg/g) and a variety of phenolic acids, including p-hydroxybenzoic acid. Similarly, Marcillo-Parra, et al.^54^ confirmed the presence of quercetin, gallic acid, and other flavonol glycosides in mango peel extracts. These phenolic compounds are known for their potent antioxidant properties, contributing to the high radical scavenging activity observed in EMPF. The co-occurrence of quercetin and p-hydroxybenzoic acid in mango peel supports their role as key bioactive constituents, reinforcing the potential of mango peel as a valuable source of natural antioxidants for functional food and nutraceutical applications.

Moreover, mango peel is particularly rich in mangiferin, quercetin, gallic acid, and other phenolic acids, which contribute to its antioxidant, antimicrobial, and anti-inflammatory properties^52^.

Based on its superior bioactive profile, EMPF was selected for nanoencapsulation using alginate, a biocompatible and biodegradable polymer widely used in food and pharmaceutical applications^55^. The nanoencapsulated formulation (N-EMPF) showed a significant increase in antioxidant activity (DPPH, RSA: 49%) despite a marked reduction in total phenolic content (80 µg GAE/mL), suggesting enhanced bioefficacy of encapsulated flavonoids, which increased to 1566 µg Rutin eq/mL (Table 3). This paradoxical improvement in antioxidant performance is attributed to the protective and controlled-release properties of the alginate matrix, which shields sensitive compounds from degradation and facilitates their gradual release at target sites. In general, nanoencapsulation acts as a physical barrier, shielding phenolic compounds from pro-oxidant molecules and environmental stressors. This protective effect helps to maintain or even enhance the antioxidant potential compared to the free (non-encapsulated) extracts. Moreover, the reduction of particle size to the nanometric scale significantly increases the surface area, facilitating more efficient interactions between phenolic compounds and free radicals, which further contributes to the improved antioxidant performance^56^. Together, these factors demonstrate that nanoencapsulation enhances functional stability, controlled release, and radical-scavenging efficiency, leading to improved antioxidant activity even when the quantifiable TPC is reduced.

**Fig. 3:**
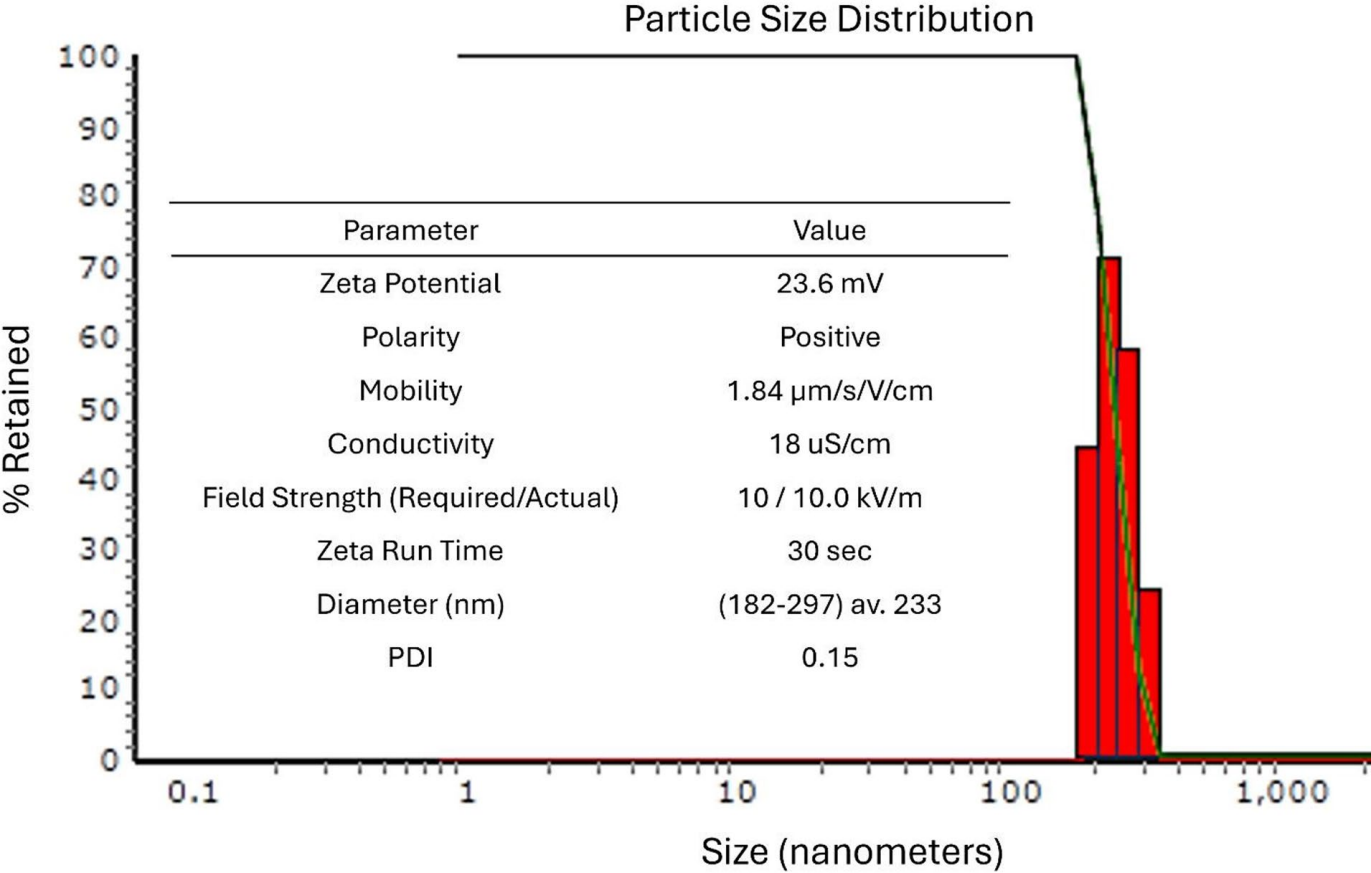
Particle size distribution and physicochemical characterization of nonencapsulated EMPF (N-EMPF).

Figure 3 confirms that the nanoparticles had an average diameter of 233 nm, a polydispersity index (PDI) of 0.15, and a Zeta potential of + 23.6 mV, which are indicative parameters for a narrow, stable, and uniform colloidal system. In agreement with the obtained results, nanoparticles in the 100–300 nm range are known to enhance gastrointestinal absorption and cellular uptake, improving the delivery and bioavailability of encapsulated compounds. The positive surface charge promotes interaction with negatively charged cell membranes, thereby enhancing cellular uptake and antioxidant activity^55^.

The encapsulation efficiency (EE%) value was calculated by measuring the amount of total polyphenols in the extract before and after loading in alginate nanospheres^32^. In addition to particle size, EE is also a very important factor in the encapsulation procedures. At a 9:1 ratio of alginate to CaCl_2_, (EE) of 56% reflects a satisfactory entrapment of bioactives within the alginate matrix, which is typical for ionic gelation techniques. Using the same alginate-to-CaCl₂ ratio, Rahnemoon, et al.^30^, reported encapsulation efficiency (EE) values ranging from 74.94% to 83.90%. However, at a mixing ratio of 6:1, the results were comparable, with EE values ranging from 57.03% to 72.92%. This difference in EE% may be due to the physicochemical properties of the formulated N-EMPF. The physicochemical stability of the N-EMPF formulation, as indicated by its mobility (1.84 μm/s/V/cm) and conductivity (18 µS/cm), aligns with the requirements for functional food and nutraceutical applications. Recent reviews emphasize that alginate-based nanocarriers are effective in enhancing the bioavailability and therapeutic potential of plant-derived bioactive components, particularly polyphenols and flavonoids^55^. In general, the integration of chemical profiling and nanoparticle characterization demonstrates that nanoencapsulation not only preserves but enhances the functional properties of mango peel extracts, offering a sustainable and innovative strategy for valorizing agro-industrial waste into high-value health-promoting products.

### Antibacterial potential of mango peel extracts

The antibacterial activity of mango peel extracts was assessed by determining their minimum inhibitory concentrations (MICs) against six common foodborne pathogenic bacteria. Before determining MICs, the antibiotic resistance profile of six pathogenic bacteria was studied against 10 antibiotics (Table 4). The sensitivity of each strain to each antibiotic was determined according to what was stated in the Clinical and Laboratory Standards Institute^57^. The results of the antibiotic susceptibility test revealed that all bacterial strains tested exhibited varying levels of resistance, with a clear prevalence of multidrug resistance (MDR). *Listeria monocytogenes* showed the highest level of resistance (8 antibiotics), followed by *Bacillus cereus*, *Campylobacter jejuni*, and *Salmonella* Typhimurium, each resistant to 7 antibiotics. In contrast, *Staphylococcus aureus* demonstrated the lowest resistance profile, being resistant to only 2 antibiotics. These findings highlight potential therapeutic challenges and underscore the importance of exploring effective natural alternatives to combat resistant strains.

**Table 4:** Antibiotic resistance profile^*^ of pathogenic bacteria against 10 antibiotics.

Antibiotics/Group	Gram-positive			Gram-negative		
	B. cereus ATCC 1076	L. monocytogenes ATCC 7644	S. aureus ATCC 653 *P*	C. jejuni ss. jejuni CCM 6214	E.coli ATCC 10,536	S. Typhimurium ATCC 14,028
Beta-lactams						
Cefadroxil (CFR-30 µg)	R	R	S	R	R	R
Flucloxacillin (FL-5 µg)	R	R	R	R	R	R
Oxacillin (OX-5 µg)	R	R	S	R	R	R
Penicillin-G (P-10 µg)	R	R	R	R	R	R
Piperacillin (PRL-100 µg)	R	R	S	R	I	R
Fluoroquinolones						
Novobiocin (NV-5 µg)	R	R	S	R	R	R
Pefloxacin (PEF-5 µg)	R	R	S	R	R	R
Aminoglycosides						
Streptomycin (S-10 µg)	S	I	I	I	I	I
Phenicols						
Chloramphenicol (C-30 µg)	S	R	S	S	S	S
Aminocoumarins						
Norfloxacin (NOR-10 µg)	S	S	S	S	I	S
Antibiotic resistance frequency Multidrug resistance (MDR)	7	8	2	7	6	7

^*^ Antibiotic resistance degree was given based on the inhibition zone (mm). Resistant (R), Intermediate (I), and Susceptible (S). Zone diameter breakpoints of antibiotics used in the present study according to the clinical and laboratory standards institute (CLSI, 2020).

As shown in Table 5, all tested extracts exhibited varying degrees of antibacterial activity, with MIC values ranging from 1 to 6 mg/mL. Notably, the ethanolic fraction (EMPF) and its encapsulated nanoparticles (N-EMPF) demonstrated the strongest antibacterial effects across most bacterial strains, with MICs as low as 1 mg/mL. Among the tested strains, the gram-positive pathogens, *Staphylococcus aureus* and *Bacillus cereus*, were the most susceptible, with MIC values of 1 mg/mL for N-EMPF. In contrast, *Campylobacter jejuni* exhibited the highest resistance, particularly against the SMPC, with a MIC of 6 mg/mL. The superior performance of EMPF and N-EMPF suggests that ethanolic extraction and fractionation enhance the concentration of bioactive compounds responsible for antibacterial activity. Several studies have linked the antimicrobial properties of mango peel extracts to their rich composition of phenolic compounds such as ellagic acid, gallic acid, tannic acid, mangiferin, and quercetin^58,59^. These compounds act through various mechanisms, including disrupting bacterial membranes and inhibiting vital enzymes. Ethanolic extracts, in particular, have shown superior efficacy compared to aqueous extracts due to their ability to solubilize a broader spectrum of bioactive compounds^58,59^. Reported MIC values in a previous study ranged between 0.2 and 2.0 mg/mL of the burnet rose ethanolic extract against bacterial strains, including *E. coli*, *S. aureus*, *B. cereus*, and *C. jejuni*^60^. These findings support our current results, where the EMPF and N-EMPF demonstrated strong antibacterial activity with MIC values (1 mg/mL), especially against *B. cereus*,* L. monocytogenes*, and *S. aureus*, confirming the relevance of mango peel polyphenols as promising natural antimicrobials.

**Table 5:** Minimum inhibitory concentration (MIC) of Mango Peel extracts against foodborne pathogenic bacteria.

Strains	MICs (mg/mL)				
	SMPC	SMPF	EMPC	EMPF	*N*- EMPF
Gram-negative bacteria					
* Campylobacter jejuni ss. Jejuni* CCM 6214	6	4	3	1	2
* Escherichia coli* ATCC 10,536	2	4	2	2	2
* Salmonella* Typhimurium ATCC 14,028	3	2	2	2	3
Gram-positive bacteria					
* Bacillus cereus* ATCC 1076	2	3	1	3	1
* Listeria monocytogenes* ATCC 7644	4	2	4	3	1
* Staphylococcus aureus* ATCC 653 P	3	4	1	1	1

*SMPC* Supercritical extracts of mango peel coarse ‎powder, *SMPF* Supercritical extracts of mango peel fine ‎powder, *EMPC* Ethanolic extract of mango peel coarse powder, *EMPF* Ethanolic extract of mango peel fine powder, *N-EMPF* Nonencapsulated EMPF.

In general, the comparison showed that the MIC values (1–6 mg/mL) for mango peel extracts were higher than those of standard antibiotics (0.01–0.1 mg/mL), which is typical for plant-based crude extracts due to their lower purity and complex phytochemical composition. Nevertheless, the extracts demonstrated measurable inhibition zones, confirming their moderate but significant antibacterial potential.

The antibacterial efficacy of mango peel extracts can be enhanced by improving extraction parameters. (solvent ratio, temperature, and time) and isolating the most potent phenolic and flavonoid fractions to improve specific bioactivity^19^. Carrier optimization through nanoencapsulation and formulation tuning could further increase solubility, cellular uptake, and compound stability. Collectively, these approaches could lower MIC values and expand the potential of mango peel bioactive for sustainable applications in food preservation and nutraceutical development.

### Antiadhesion and antibiofilm activities of mango peel extracts

**Fig. 4:**
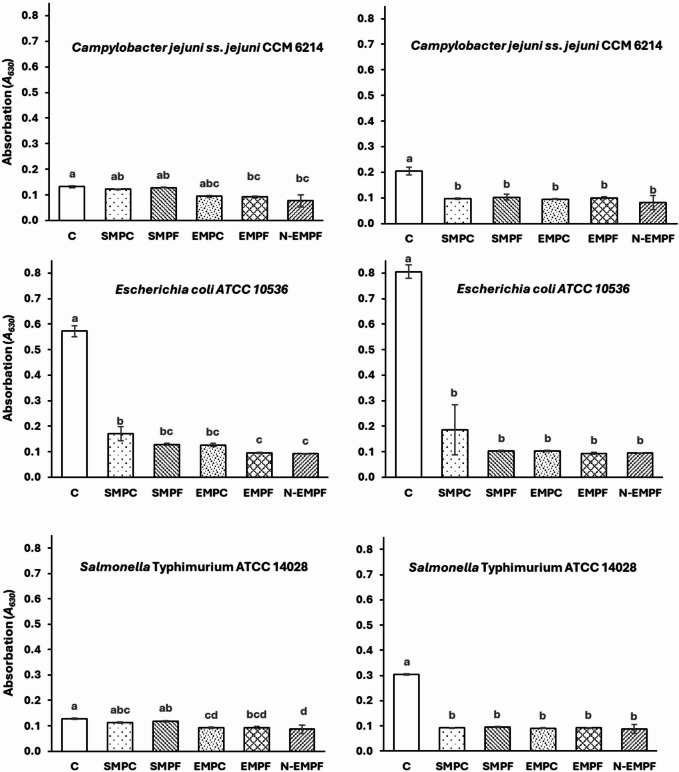
Anti-adhesion (Left) and anti-biofilm (Right) effects of mango peel extracts against gram-negative pathogenic bacteria. The mango peel extracts were obtained through supercritical CO2 extraction of mango peel coarse particles (SMPC), fine particles (SMPF), ethanolic extraction of mango peel coarse particles (EMPC) and fine particles (EMPF), and encapsulated EMPF nanoparticles (N-EMPF). Values are means (*n = 3*) ± SD. Columns of each strain with different small letters differ significantly (*P < 0.05*).

The adhesion and antibiofilm effects of the prepared extracts were studied on Gram-positive and Gram-negative bacteria. Figure 4 shows the effect of the extracts on three Gram-negative bacteria. All extracts had a significant (*p < 0.05*) antiadhesion effect on *Escherichia coli*, while the adhesion ability of *Campylobacter jejuni* and *Salmonella* Typhimurium was not significantly affected. On the other hand, all extracts had a significant antibiofilm effect on all studied strains, but the effect was more significant on *E. coli*. The ethanolic mango peel extracts, particularly from fine particles (EMPF), showed strong antiadhesion and antibiofilm activities against *E. coli *(Fig. 4). Supercritical CO₂ extracts (SMPC and SMPF) demonstrated moderate antiadhesion ‎and antibiofilm effects on *E. coli* compared to other extracts.

**Fig. 5:**
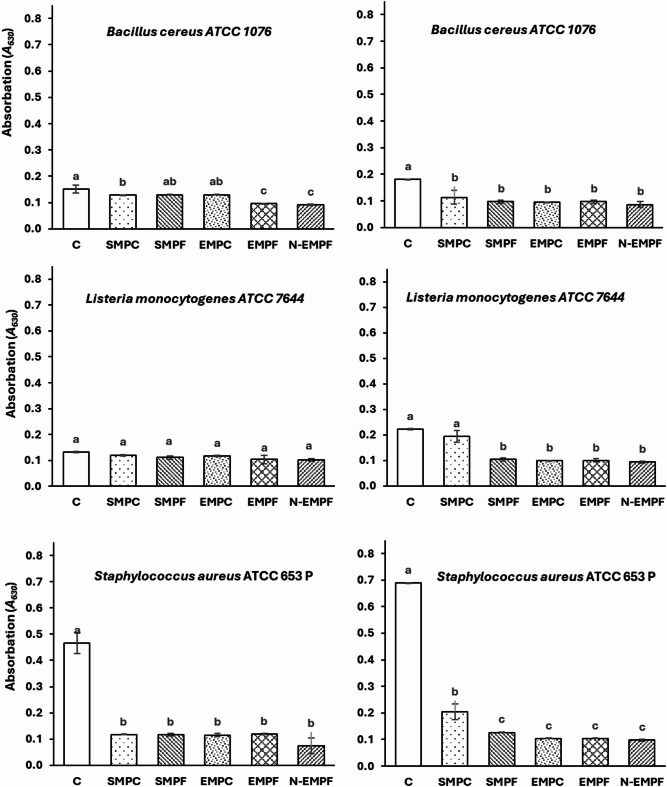
Anti-adhesion (Left) and anti-biofilm (Right) effects of mango peel extracts against gram-negative pathogenic bacteria. The mango peel extracts were obtained through supercritical CO2 extraction of mango peel coarse particles (SMPC), fine particles (SMPF), ethanolic extraction of mango peel coarse particles (EMPC) and fine particles (EMPF), and encapsulated EMPF nanoparticles (N-EMPF). Values are means (*n = 3*) ± SD. Columns of each strain with different small letters differ significantly (*P < 0.05*).

Figure 5 shows that *Staphylococcus aureus* was the most affected Gram-positive bacterium by all the extracts prepared in the current study. The ability of *Staphylococcus aureus* to adhere and form biofilms was significantly reduced after treatment with these extracts. The ability of *Bacillus cereus* and *Listeria monocytogenes* to form biofilms was significantly affected, but at lower values than that of *Staphylococcus aureus*. SMPC had less antibiofilm effect on Gram-positive bacteria compared to other extracts. Generally, the nanoencapsulated ethanolic extract (N-EMPF) exhibited the highest significant (*p < 0.05*) antiadhesion and antibiofilm activity among most treatments (Figs. 4 and 5).

The potent antibiofilm activity observed in mango peel extract (especially EMPF) is primarily ‎attributed to its high content of polar bioactive compounds, including phenolic acids, flavonoids, and gallic acid derivatives. These compounds are efficiently extracted using ethanol, particularly when fine particle sizes are employed, which enhances surface area and solvent penetration, thereby improving extraction efficiency^61^. HPLC analysis of EMPF confirmed the presence of quercetin and p-hydroxybenzoic acid as major phenolic constituents (supplemented data, S2), both of which are known for their strong antioxidant and antimicrobial properties. These findings are consistent with previous studies reporting broad-spectrum antibiofilm activity of mango peel phenolics against pathogens such as *Staphylococcus aureus*, *Escherichia coli*, and *Bacillus cereus*^20^. The antimicrobial efficacy of EMPF is closely linked to the ability of its phenolic constituents to disrupt bacterial membranes and inhibit essential enzymes^15^. Notably, quercetin has been shown to inhibit bacterial adhesion and biofilm formation by interfering with quorum sensing, reducing motility, and suppressing virulence gene expression^62^. Similarly, p-hydroxybenzoic acid contributes to antibiofilm activity by disrupting membrane integrity and inhibiting extracellular polymeric substance (EPS) production, which is essential for biofilm matrix development^63^. Furthermore, nanoencapsulation of these bioactive compounds significantly enhances their stability, solubility, and bioavailability, allowing for improved penetration into bacterial cells and biofilms. This controlled release mechanism improves overall antimicrobial and antibiofilm efficacy^64^. These findings underscore the potential of mango peel phenolics, particularly quercetin and p-hydroxybenzoic acid, as sustainable and effective agents in combating biofilm-associated infections.

### Cytotoxicity assay of EMPF and N-EMPF

**Fig. 6:**
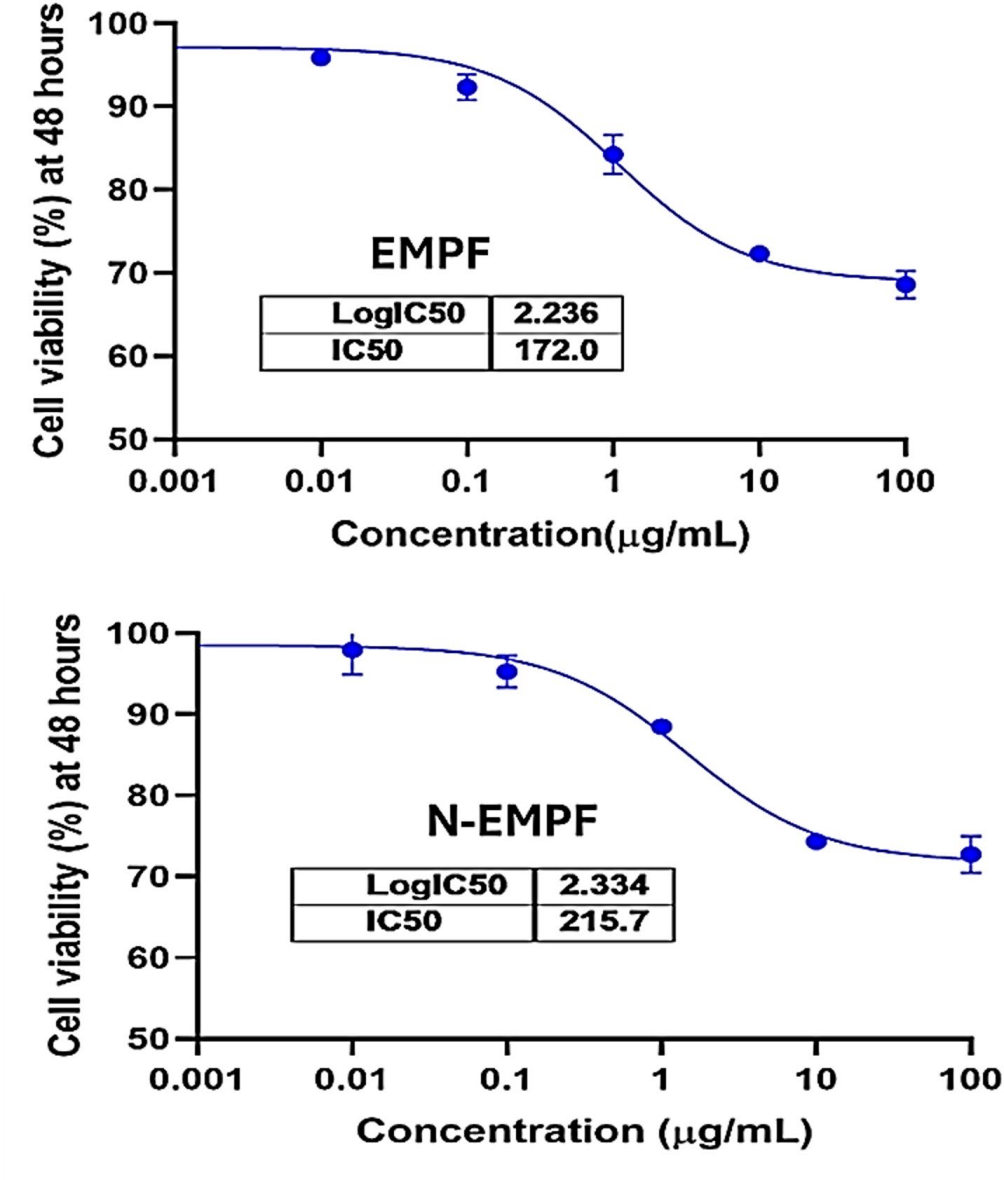
Cytotoxic effect (IC_50_) of ethanolic extract of mango peel fine powder (EMPF) and encapsulated nanoparticles (N-EMPF) on normal oral epithelial cells (h-OECs).

The cytotoxicity of both EMPF and N-EMPF was studied, which proved to be the best extracts in terms of antioxidant, anti-adhesion, antimicrobial, and anti-biofilm activity in previous experiments. As shown in Table 6, both EMPF and N-EMPF induced a dose-dependent decrease in cell viability after 48 h of exposure. EMPF demonstrated a gradual decline in viability from 95.87% at 0.1 µg/mL to 68.59% at 100 µg/mL, while N-EMPF exhibited slightly higher safety, with viability ranging from 97.92% to 72.73% across the same concentration range. The calculated half-maximal inhibitory concentration (IC₅₀) was > 100 µg/mL for both extracts, indicating low cytotoxicity under the tested conditions (Fig. 6). The IC₅₀ value was 172.0 and 215.7 µg/mL for EMPF and N-EMPF, respectively. The IC₅₀ value for both extracts was > 100 µmol/mL, meaning that the concentration required to induce a 50% cytotoxic effect was not reached within the tested range, as an indicator of favorable safety. According to ISO 10,993–5 guidelines, materials with IC₅₀ values above certain thresholds (e.g., ≥ 90 µg/mL) are classified as non-cytotoxic, supporting their safety for further development^65^. Therefore, the EMPF and N-EMPF formulations demonstrate a favorable safety profile consistent with international standards for nutraceutical and food-grade materials.

**Table 6:** MTT assay results for normal oral epithelial cells (h-OECs) treated with different concentrations of EMPF and N-EMPF for 48 h.

Extract	Average cell viability%					
	Tested concentration (µg/mL)					
	HNO97	0.01	0.1	1	10	100
EMPF	100.00%	95.87%	92.31%	84.22%	72.31%	68.59%
N-EMPF	100.00%	97.92%	95.29%	88.46%	74.33%	72.73%

*MTT* 4,5-dimethylthiazol-2-yl)−2,5-diphenyltetrazolium bromide, *EMPF* Ethanolic extract of mango peel fine powder, *N-EMPF* Nonencapsulated EMPF.

Mahmoud, et al.^66^ conducted a study on the anticancer and antioxidant activities of ethanolic extracts and semi-purified fractions from mango and guava seeds. They reported that the ethanolic mango extract exhibited low cytotoxicity, with IC₅₀ values exceeding 100 µg/mL in most cases, indicating a favorable safety profile.

In general, both extracts were relatively safe and did not cause a significant reduction in cell viability, even at the highest tested concentration. However, N-EMPF exhibited a lower level of cytotoxicity (higher cell viability) across all concentrations, indicating an improvement in biological safety following nano-formulation.

**Fig. 7:**
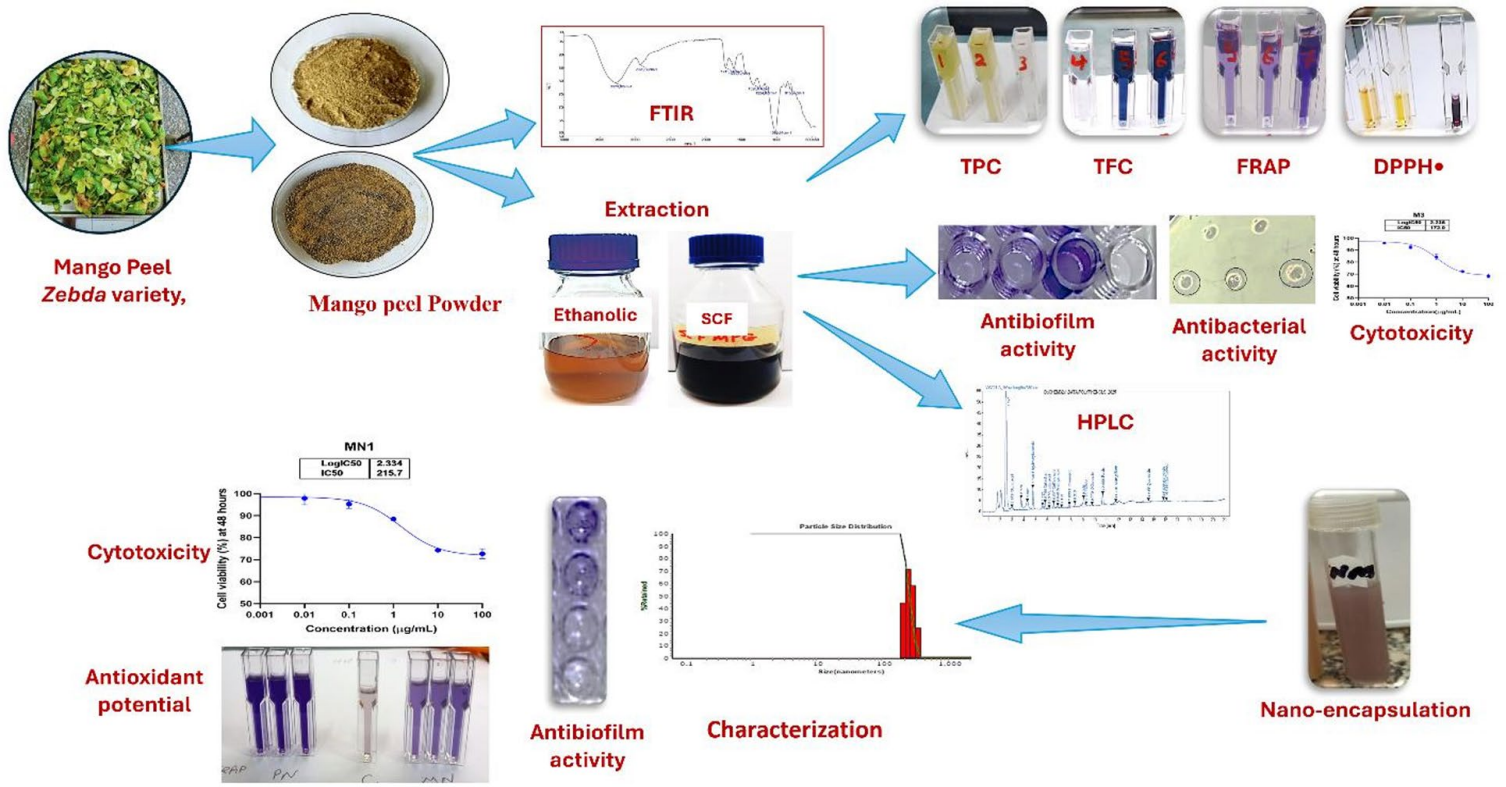
Schematic diagram summarizing all technological steps, including extraction, nano-encapsulation, and evaluation of the produced mango peel extracts.

At the end of this work, we can summarize the overall technological steps, including extraction, nanoencapsulation, and evaluation of the produced mango peel extracts in Fig. 7.

## Conclusion

This study highlights the potential of mango peel waste as a sustainable and valuable source of bioactive compounds with diverse functional applications. Ethanolic extraction from fine mango peel powder yielded extracts rich in phenolics and flavonoids. Both ethanolic mango peel extracts (EMPF) and their nanoencapsulated forms (N-EMPF) exhibit potent antibacterial, antiadhesion, and antibiofilm activity against multidrug-resistant (MDR) bacteria, including *Listeria monocytogenes*, *Bacillus cereus*, and *Staphylococcus aureus*. Furthermore, nanoencapsulation enhanced the extract’s bioactivity and stability without compromising safety, as demonstrated by cytotoxicity assays. Overall, mango peel-derived extracts and their nano-formulations show strong promise as safe, eco-friendly alternatives for applications in the food industry. Future research is needed on scaling up the extraction and encapsulation processes for industrial applications.

## Supplementary Information

Below is the link to the electronic supplementary material.

Supplementary Material 1

Supplementary Material 2

## Data Availability

All data from the authors are available upon reasonable request.

## References

[1] Bian, X. et al. Functionalized Polyvinyl alcohol nanofibers with visible light-triggered antibacterial and ethylene scavenging capabilities for food packaging. *Food Packaging Shelf Life*. **36**, 101056. 10.1016/j.fpsl.2023.101056 (2023).

[2] Mitrea, L. et al. In *In Fruit and vegetable waste utilization and sustainability* (eds Mandavgane, S. A. et al.) 43–76 (Academic, 2023).

[3] Hussain, A. et al. Exploration of underutilized chayote fractions following drying and extraction. *Food Chem.* **465**, 142129. 10.1016/j.foodchem.2024.142129 (2025). 39579399 10.1016/j.foodchem.2024.142129

[4] Emam, M., Soliman, M. M. H., Tantawy, M. A., El-Ansari, M. A. & Seif, M. Pentagalloyl glucose and gallotannins from *mangifera indica* L. seed kernel extract as a promising antifungal feed additive, *in vitro*, and *in Silico* studies. *J. Stored Prod. Res.* **107**, 102354. 10.1016/j.jspr.2024.102354 (2024).

[5] Nyangena, I. O., Owino, W. O., Imathiu, S. & Ambuko, J. Effect of pretreatments prior to drying on antioxidant properties of dried Mango slices. *Sci. Afr.* **6**, e00148. 10.1016/j.sciaf.2019.e00148 (2019). 10.1007/s13197-019-03857-9 PMC6675852 31413411

[6] Kumar, A., Kumar, A., Gehlot, R., Singh, D. & Chaudhary, T. In *In adding value to fruit wastes* (eds Bangar, S. P., Parmjit, S. et al.) 261–290 (Academic, 2024).

[7] Cárdenas-Hernández, E. et al. From agroindustrial waste to nutraceuticals: potential of Mango seed for sustainable product development. *Trends Food Sci. Technol.* **154**, 104754. 10.1016/j.tifs.2024.104754 (2024).

[8] Karmakar, R. et al. Valorization of food waste stream by Harnessing bioactive compounds: A comprehensive review on the process, challenges and solutions. *Food Bioscience*. **69**, 106833. 10.1016/j.fbio.2025.106833 (2025).

[9] Ojeda, G. A., Sgroppo, S. C., Sánchez-Moreno, C. & de Ancos, B. Mango ‘criollo’ by-products as a source of polyphenols with antioxidant capacity. Ultrasound assisted extraction evaluated by response surface methodology and HPLC-ESI-QTOF-MS/MS characterization. *Food Chem.* **396**, 133738. 10.1016/j.foodchem.2022.133738 (2022). 35872495 10.1016/j.foodchem.2022.133738

[10] Nitisuk, P., Wanyo, P., Chamsai, T. & Charoenjit, K. Sustainable valorization of tropical fruit peels for sustainable production of natural antioxidants and functional food ingredients. *Sustainable Food Technol.* **3**, 1189–1202. 10.1039/d4fb00371c (2025).

[11] RedCorn, R., Fatemi, S. & Engelberth, A. S. Comparing End-Use potential for industrial Food-Waste sources. *Engineering* **4**, 371–380. 10.1016/j.eng.2018.05.010 (2018).

[12] Pasrija, P., Girdhar, M., Kumar, M., Arora, S. & Katyal, A. Endophytes: an unexplored treasure to combat multidrug resistance. *Phytomedicine Plus*. **2**, 100249. 10.1016/j.phyplu.2022.100249 (2022).

[13] Gupta, P. D. & Birdi, T. J. Development of botanicals to combat antibiotic resistance. *J. Ayurveda Integr. Med.* **8**, 266–275. 10.1016/j.jaim.2017.05.004 (2017). 28869082 10.1016/j.jaim.2017.05.004 PMC5747506

[14] Trinchera, M. et al. Antimicrobials in livestock farming and resistance: public health implications. *Antibiotics* **14**, 606 (2025). 40558196 10.3390/antibiotics14060606 PMC12190098

[15] Semangoen, T. et al. Antimicrobial efficacy of mangosteen (*Garcinia mangostana*) Peel extracts in airborne microbial control within livestock farming environments. *Microb. Pathog.* **204**, 107618. 10.1016/j.micpath.2025.107618 (2025). 40254079 10.1016/j.micpath.2025.107618

[16] Cazón, P., Mateus, A. R. & Silva, A. S. Advances in active packaging using natural biopolymers and fruit by-products for enhanced food preservation. *Food Res. Int.* **213**, 116439. 10.1016/j.foodres.2025.116439 (2025). 40436549 10.1016/j.foodres.2025.116439

[17] Macías-Sánchez, M. D. et al. Supercritical fluid extraction of carotenoids and chlorophyll a from *Synechococcus* Sp. *J. Supercrit. Fluids*. **39**, 323–329. 10.1016/j.supflu.2006.03.008 (2007).

[18] Fernández-Ponce, M. T., Casas, L., Mantell, C. & Rodríguez, M. Martínez de La Ossa, E. Extraction of antioxidant compounds from different varieties of *Mangifera indica* leaves using green technologies. *J. Supercrit. Fluids*. **72**, 168–175. 10.1016/j.supflu.2012.07.016 (2012).

[19] Giri, S., Kshirod Kumar, D., Bhagya Raj, G. V. S. & Kovács, B. Ayaz Mukarram, S. Ultrasound assisted phytochemical extraction of persimmon fruit peel: integrating ANN modeling and genetic algorithm optimization. *Ultrason. Sonochem.* **102**, 106759. 10.1016/j.ultsonch.2024.106759 (2024). 38211494 10.1016/j.ultsonch.2024.106759 PMC10825330

[20] Abdelmontaleb, H. S., Abdelmeged, D. A., Hamdy, S. M., Hammam, M. G. & Ebid, W. M. A. Exploring the potential of using pomegranate and Mango Peel powders as natural food additives targeting safety of white soft cheese. *Int. J. Food Microbiol.* **434**, 111158. 10.1016/j.ijfoodmicro.2025.111158 (2025). 40106872 10.1016/j.ijfoodmicro.2025.111158

[21] Mohlamonyane, M. J., Adeyemi, J. O. & Fawole, O. A. Pomegranate fruit peel: A sustainable bioresource in food preservation. *Food Bioscience*. **62**, 105532. 10.1016/j.fbio.2024.105532 (2024).

[22] Nallamuthu, I., Khanum, F., Fathima, S. J., Patil, M. M. & Anand, T. In *In Nutrient Delivery* (ed. Grumezescu, A. M.) 619–651 (Academic, 2017).

[23] Sharaf, A., Ashour, M., Zaky, A. A. & Y, E.-F. & Utilization of Mango peels as a source of polyphenolic antioxidants. *Curr. Sci. Int.* **5**, 529–542 (2016).

[24] VDLUFA & Methods Book, I. *Soil Analysis (1st – 6th Supplement Delivery)* (VDLUFA-Verlag Darmstadt, 1991).

[25] Gautam, R. K., Mudhoo, A. & Chattopadhyaya, M. C. Kinetic, equilibrium, thermodynamic studies and spectroscopic analysis of Alizarin red S removal by mustard husk. *J. Environ. Chem. Eng.* **1**, 1283–1291. 10.1016/j.jece.2013.09.021 (2013).

[26] Dorta, E., Lobo, M. G. & Gonzalez, M. Reutilization of Mango byproducts: study of the effect of extraction solvent and temperature on their antioxidant properties. *J. Food Sci.* **77**, C80–88. 10.1111/j.1750-3841.2011.02477.x (2012). 22132766 10.1111/j.1750-3841.2011.02477.x

[27] Yilmaz, E. E., Özvural, E. B. & Vural, H. Extraction and identification of proanthocyanidins from grape seed (*Vitis Vinifera*) using supercritical carbon dioxide. *J. Supercrit. Fluids*. **55**, 924–928. 10.1016/j.supflu.2010.10.046 (2011).

[28] Attallah, E. R., Hamdy Abdelwahed, M. & Abo-Aly, M. Development and validation of multi-residue method for determination of 412 pesticide residues in cotton fiber using GC-MS/MS and LC-MS/MS. *J. Text. Inst.* **109**, 46–63 (2018).

[29] Paques, J. P., van der Linden, E., van Rijn, C. J. M. & Sagis, L. M. C. Alginate submicron beads prepared through w/o emulsification and gelation with CaCl_2_ nanoparticles. *Food Hydrocoll.* **31**, 428–434. 10.1016/j.foodhyd.2012.11.012 (2013).

[30] Rahnemoon, P., Sarabi-Jamab, M., Bostan, A. & Mansouri, E. Nano-encapsulation of pomegranate (*Punica granatum* L.) Peel extract and evaluation of its antimicrobial properties on coated chicken meat. *Food Bioscience*. **43**, 101331. 10.1016/j.fbio.2021.101331 (2021).

[31] Gaber Ahmed, G. H., Fernández-González, A. & Díaz García, M. E. Nano-encapsulation of grape and Apple pomace phenolic extract in Chitosan and soy protein via nanoemulsification. *Food Hydrocoll.* **108**, 105806. 10.1016/j.foodhyd.2020.105806 (2020).

[32] Saikia, S., Mahnot, N. K. & Mahanta, C. L. Optimisation of phenolic extraction from *Averrhoa Carambola* pomace by response surface methodology and its microencapsulation by spray and freeze drying. *Food Chem.* **171**, 144–152. 10.1016/j.foodchem.2014.08.064 (2015). 25308654 10.1016/j.foodchem.2014.08.064

[33] Hazekawa, M., Nishinakagawa, T., Kawakubo-Yasukochi, T. & Nakashima, M. Evaluation of IC_50_ levels immediately after treatment with anticancer reagents using a real-time cell monitoring device. *Exp. Ther. Med.* **18**, 3197–3205. 10.3892/etm.2019.7876 (2019). 31555392 10.3892/etm.2019.7876 PMC6755379

[34] Singleton, V. L., Orthofer, R. & Lamuela-Raventós, R. M. In *Methods in Enzymology.* **299** 152–178 (Academic Press, 1999).

[35] Chang, C. C., Yang, M. H., Wen, H. M. & Chern, J. C. Estimation of total flavonoid content in propolis by two complementary colometric methods. *J. food drug analy.* **10**, 3 (2002).

[36] Brand-Williams, W., Cuvelier, M. E. & Berset, C. Use of a free radical method to evaluate antioxidant activity. *LWT - Food Sci. Technol.* **28**, 25–30. 10.1016/S0023-6438(95)80008-5 (1995).

[37] Benzie, I. F. F. & Strain, J. J. The ferric reducing ability of plasma (FRAP) as a measure of antioxidant power: the FRAP assay. *Anal. Biochem.* **239**, 70–76. 10.1006/abio.1996.0292 (1996). 8660627 10.1006/abio.1996.0292

[38] Manandhar, S., Luitel, S. & Dahal, R. K. In vitro antimicrobial activity of some medicinal plants against human pathogenic bacteria. *J. Tropic. Med.* **2019**, 1895340 (2019). 10.1155/2019/1895340 PMC6466868 31065287

[39] Bauer, A., Kirby, W., Sherris, J. C. & Turck, M. Antibiotic susceptibility testing by a standardized single disk method. *Am. J. Clin. Pathol.* **45**, 493–496 (1966). 5325707

[40] Christensen, G. D. et al. Adherence of coagulase-negative Staphylococci to plastic tissue culture plates: a quantitative model for the adherence of Staphylococci to medical devices. *J. Clin. Microbiol.* **22**, 996–1006 (1985). 3905855 10.1128/jcm.22.6.996-1006.1985 PMC271866

[41] Stepanović, S., Vuković, D., Dakić, I. & Savić, B. Švabić-Vlahović, M. A modified microtiter-plate test for quantification of Staphylococcal biofilm formation. *J. Microbiol. Methods*. **40**, 175–179 (2000). 10699673 10.1016/s0167-7012(00)00122-6

[42] Mastoor, S. et al. Analysis of the antimicrobial and anti-biofilm activity of natural compounds and their analogues against *Staphylococcus aureus* isolates. *Molecules* **27**, 6874 (2022). 36296467 10.3390/molecules27206874 PMC9610881

[43] Marçal, S. & Pintado, M. Mango peels as food ingredient / additive: nutritional value, processing, safety and applications. *Trends Food Sci. Technol.* **114**, 472–489. 10.1016/j.tifs.2021.06.012 (2021).

[44] Sánchez-Camargo, A. et al. Valorisation of mango peel: Proximate composition, supercritical fluid extraction of carotenoids, and application as an antioxidant additive for an edible oil. *J. Supercritical Fluids* **152**, 104574. 10.1016/j.supflu.2019.104574 (2019).

[45] Fekhar, M., Daghbouche, Y., Bouzidi, N. & El Hattab, M. Rapid assessment of smokeless tobacco quality parameters using ATR-FT-MIR spectroscopy: comparison of analytical/mathematical and machine learning approaches. *Microchem. J.* **201**, 110670. 10.1016/j.microc.2024.110670 (2024).

[46] Ramezanzadeh, M., Bahlakeh, G. & Ramezanzadeh, B. Study of the synergistic effect of *Mangifera indica* leaves extract and zinc ions on the mild steel corrosion Inhibition in simulated seawater: computational and electrochemical studies. *J. Mol. Liq.* **292**, 111387. 10.1016/j.molliq.2019.111387 (2019).

[47] Putra, N. R. et al. Influence of particle size in supercritical carbon dioxide extraction of roselle (*Hibiscus sabdariffa*) on bioactive compound recovery, extraction rate, diffusivity, and solubility. *Sci. Rep.* **13**, 10871. 10.1038/s41598-023-32181-8 (2023). 37407592 10.1038/s41598-023-32181-8 PMC10322909

[48] Li, H., Zhang, Z., Tang, S., Li, Y. & Zhang, Y. Ultrasonically assisted acid extraction of manganese from slag. *Ultrason. Sonochem.* **15**, 339–343. 10.1016/j.ultsonch.2007.07.010 (2008). 17888712 10.1016/j.ultsonch.2007.07.010

[49] Saleh, A. K., Aboelghait, K. M., El-Fakharany, E. M. & El-Gendi, H. Multifunctional engineering of mangifera indica L. peel extract-modified bacterial cellulose hydrogel: unveiling novel strategies for enhanced heavy metal sequestration and cytotoxicity evaluation. *Int. J. Biol. Macromol.* **278**, 134874. 10.1016/j.ijbiomac.2024.134874 (2024). 39168196 10.1016/j.ijbiomac.2024.134874

[50] Kučuk, N., Primožič, M., Kotnik, P., Knez, Ž. & Leitgeb, M. Mango peels as an industrial by-product: a sustainable source of compounds with antioxidant, enzymatic, and antimicrobial activity. *Foods* **13**, 553 (2024). 38397530 10.3390/foods13040553 PMC10888073

[51] Chammam, A. et al. Supercritical CO_2_ extraction of bioactives from *P. halepensis* petals: process modeling, mass transfer, and bioactivity characterization. *J. Supercrit. Fluids*. **225**, 106701. 10.1016/j.supflu.2025.106701 (2025).

[52] Choudhary, P. et al. Mango seed kernel: A bountiful source of nutritional and bioactive compounds. *Food Bioprocess Technol.* **16**, 289–312. 10.1007/s11947-022-02889-y (2023).

[53] Suleria, H. A. R., Barrow, C. J. & Dunshea, F. R. Screening and characterization of phenolic compounds and their antioxidant capacity in different fruit peels. *Foods* **9**, 1206. 10.3390/foods9091206 (2020). 32882848 10.3390/foods9091206 PMC7556026

[54] Marcillo-Parra, V., Tupuna-Yerovi, D. S., Molina, M., Balladares, K. A. H. & Ruales, J. UPLC-PDA phenolic compounds profile of Mango Peel extracts obtained using different solvents and ultrasound-assisted extraction. *Food. Anal. Methods*. **17**, 1466–1472. 10.1007/s12161-024-02651-4 (2024).

[55] Saadi, S. et al. A review on trends in microencapsulation of bioactive compounds: coating materials, design, and applications. *Eur. Food Res. Technol.* **249**, 3123–3139. 10.1007/s00217-023-04354-2 (2023).

[56] Abushal, S. A., Elhendy, H. A. & Soliman, T. N. Nanoencapsulation of *Thyme oleoresin*: A novel strategy to enhance bioactive ingredients’ bioaccessibility in functional UF-Labneh and prolong shelf life. *J. Future Foods*. 10.1016/j.jfutfo.2025.03.013 (2025).

[57] CLSI. Performance standards for antimicrobial susceptibility testing. *Clinical and Laboratory Standards Institute CLSI supplement M100*. (Wayne, 2020).

[58] Brito, T. B. N. et al. Antimicrobial, antioxidant, volatile and phenolic profiles of cabbage-stalk and pineapple-crown flour revealed by GC-MS and UPLC-MSE. *Food Chem.* **339**, 127882. 10.1016/j.foodchem.2020.127882 (2021). 32889131 10.1016/j.foodchem.2020.127882

[59] Ribeiro, A. C. B. et al. From Mango by-product to food packaging: Pectin-phenolic antioxidant films from Mango peels. *Int. J. Biol. Macromol.* **193**, 1138–1150. 10.1016/j.ijbiomac.2021.10.131 (2021). 34717979 10.1016/j.ijbiomac.2021.10.131

[60] Findik, B. T. et al. Phytochemical profile, enzyme inhibition, antioxidant, and antibacterial activity of *Rosa Pimpinellifolia* L.: A comprehensive study to investigate the bioactivity of different parts (whole fruit, pulp, and seed part) of the fruit. *Food Chem.* **455**, 139921. 10.1016/j.foodchem.2024.139921 (2024). 38843718 10.1016/j.foodchem.2024.139921

[61] Ashong, G. W., Darko, C. E., Pappoe, E., Ababio, B. A. & Kwaansa-Ansah, E. E. Exploration of the phytochemical evaluation, chemical profile, and antimicrobial activities of cashew nut shell oil, a potential medicinal plant for various applications. *Pharmacol. Res. - Nat. Prod.* **8**, 100291. 10.1016/j.prenap.2025.100291 (2025).

[62] Gopu, V., Meena, C. K. & Shetty, P. H. Quercetin influences quorum sensing in food borne bacteria: In-Vvtro and In-silico evidence. *PLOS ONE*. **10**, e0134684. 10.1371/journal.pone.0134684 (2015). 26248208 10.1371/journal.pone.0134684 PMC4527846

[63] Campbell, M. et al. 4-Ethoxybenzoic acid inhibits *Staphylococcus aureus* biofilm formation and potentiates biofilm sensitivity to Vancomycin. *Int. J. Antimicrob. Agents*. **56**, 106086. 10.1016/j.ijantimicag.2020.106086 (2020). 32663508 10.1016/j.ijantimicag.2020.106086

[64] Saborirad, S., Baghaei, H. & Hashemi-Moghaddam, H. Optimizing the ultrasonic extraction of polyphenols from Mango Peel and investigating the characteristics, antioxidant activity and storage stability of extract nanocapsules in maltodextrin/whey protein isolate. *Ultrason. Sonochem.* **103**, 106778. 10.1016/j.ultsonch.2024.106778 (2024). 38262176 10.1016/j.ultsonch.2024.106778 PMC10832609

[65] Al-Rawe, R. A., Alsheekhly, B., Al-Rammahi, H. M. & Alhasan, A. Ma’amor, A. Harnessing Antarctic Krill for nano-fluorapatite synthesis: A novel biomimetic approach for dental applications. *J. Indian Chem. Soc.* **102**, 101845. 10.1016/j.jics.2025.101845 (2025).

[66] Mahmoud, M., Mohamed, E. M., Aboul-Enein, A. M., Diab, A. A. & Shalaby, E. A. Anticancer and antioxidant activities of ethanolic extract and semi-purified fractions from guava and Mango seeds. *Biomass Convers. Biorefinery*. **14**, 20153–20169. 10.1007/s13399-023-04216-7 (2024).

