# Supplementary material for: Sustainable valorization of mango peel waste by extracting bioactive compounds for functional applications
Source: Sci Rep. 2025 Dec 7;15:43283. doi: 10.1038/s41598-025-28141-z (PMC12686472; doi:10.1038/s41598-025-28141-z)
Supplement: Supplementary file 1 — Supplementary Material 1 [file 41598_2025_28141_MOESM1_ESM.pdf]

S1: Determination of Pesticide Residues Using LC-MS/MS in Mango Peel Extracts (EMPC, EMPF, SMPC, and SMPF) Categorized by Chemical Class.

Pesticide Chemical Class*	Count of Pesticides	Pesticide Chemical Class*	Count of Pesticides	Pesticide Chemical Class*	Count of Pesticides	Pesticide Chemical Class*	Count of Pesticides
Acylalanine	1	Botanical	2	Morpholine	5	Pyrimidine	3
Acylalanine / Xylylalanine	1	Carbamate	40	Neonicotinoid	6	Pyrimidinol	2
Acylpicolide	1	Carboxamide	1	Nereistoxin	1	Pyrimidinyloxybenzoic	1
Amide	4	Carboxamide / Oxathiin	1	Organochlorine	3	Quinoline	1
Amine	1	Chloroacetamide	1	Organophosphorus	71	Quinolinecarboxylic acid	1
Anilide	4	Chloroacetamide / Chloroacetanilide	1	Oxadiazine	1	Reductase	1
Anilide / Oxathiin	1	Chloroacetamide /Chloroacetanilide	3	Oxadiazole	2	Spinosyn	1
Anilide / Phenylamide	1	Coumarin	1	Oxathiin	1	Strobilurin	4
Anilinopyrimidine	2	Cyclohexanedione	1	Oxathiin / Anilide	1	Strobilurin / Strobin	1
Aromatic hydrocarbon	1	Cyclohexanedione oxime	3	Oxyacetamide / Anilide	1	Sulfonamide	1
Aryloxyphenoxy propionic acid	3	Diacylhydrazine	3	Phenylpyrazole	1	Sulphamide	1
Aryloxyphenoxypropionic acid/ester	4	Dicarboximide	2	Phosphorothioate	2	Tetrazine	1
Avermectin	2	Dicarboximide / Strobilurin	1	Phthalimide	1	Tetronic acid	1
Azole	25	Dinitroaniline	4	Piperazine	1	Triazine	16
Benzamide	2	Guanidine	1	Pyrazole	2	Triazinone	3
Benzamide / Oxazole	1	Imidazole	3	Pyrethroid	10	Triazolopyrimidine	3
Benzimidazole	2	Imidazolinone	2	Pyridazinone	1	unclassified	18
Benzofuran	1	Isoxazolidinone	1	Pyridinecarboxamide	1	Uracil	2
Benzotriazine	1	Juvenile hormone mimic	1	Pyridinecarboxylic acid/Pyridinecarboxamide	1	Urea derivatives	29
Benzoylurea	1	Keto-enol	1	Pyridinecarboxylic acids/ester	3		

* All categories of chemical pesticides mentioned are Not detect.
