# Supplementary material for: Sustainable valorization of mango peel waste by extracting bioactive compounds for functional applications
Source: Sci Rep. 2025 Dec 7;15:43283. doi: 10.1038/s41598-025-28141-z (PMC12686472; doi:10.1038/s41598-025-28141-z)
Supplement: Supplementary file 2 — Supplementary Material 2 [file 41598_2025_28141_MOESM2_ESM.pdf]

S2 : Quantitative Analysis of Polyphenolic Compounds of Ethanolic Mango Peel Extract Using High Performance Liquid Chromatography (HPLC) at 280 nm.

Name	Expected RT (min)	RT (min)	Area	Amount (mg/L)
Gallic acid	2.9	2.98	22.4301	4.459
P hydroxybenzoic	4.8	4.76	56.3088	28.684
Catechin	5.7	5.79	16.2776	15.813
Vanillic acid	6.2	6.13	4.4536	1.479
Caffeic acid	6.4	6.51	11.802	2.747
Syringic acid	6.941	6.85	7.7342	1.473
P Coumaric	7.9	7.84	8.5374	1.264
Ferulic	9.27	9.26	5.912	4.478
OCumaric	9.55	9.78	2.4751	0.291
Rutin	10.9	10.67	37.643	10.84
Mangiferin	11.8	11.78	17.2537	6.969
Quercetin	14.405	14.55	29.9161	63.754
Apigenin	16.28	15.81	7.0937	0.213
Kaempferol	16.06	16.07	5.8759	1.642

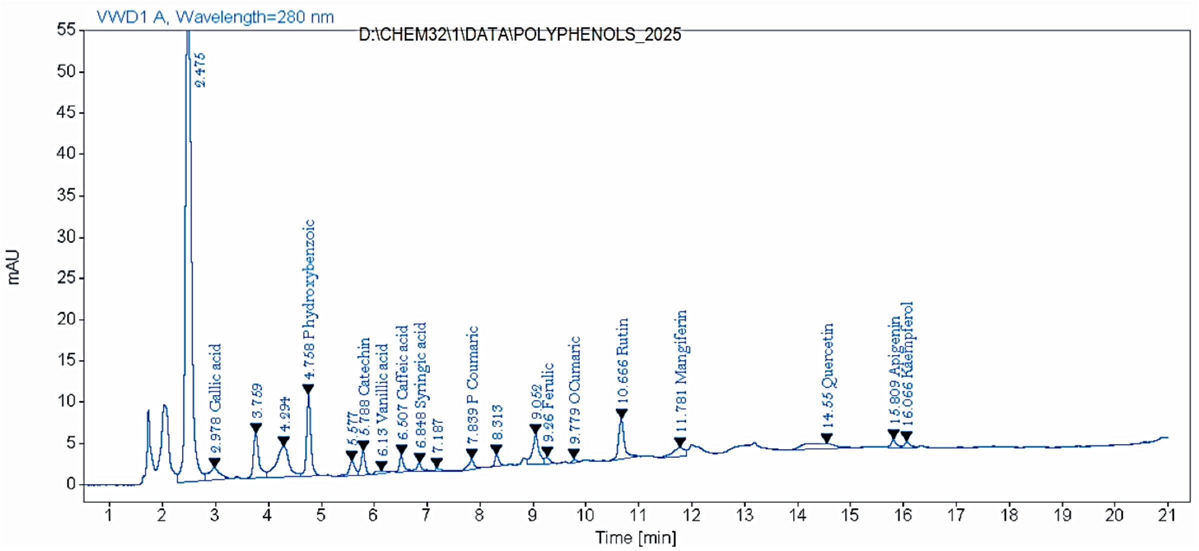

Figure : Quantitative Analysis of Polyphenolic Compounds of Ethanolic Mango Peel Extract Using High Performance Liquid Chromatography (HPLC) at 280 nm.
